# Tempo and Mode of Transposable Element Activity in Drosophila

**DOI:** 10.1371/journal.pgen.1005406

**Published:** 2015-07-17

**Authors:** Robert Kofler, Viola Nolte, Christian Schlötterer

**Affiliations:** Institut für Populationsgenetik, Vetmeduni Vienna, Wien, Austria; Stanford University, United States

## Abstract

The evolutionary dynamics of transposable element (TE) insertions have been of continued interest since TE activity has important implications for genome evolution and adaptation. Here, we infer the transposition dynamics of TEs by comparing their abundance in natural *D. melanogaster* and *D. simulans* populations. Sequencing pools of more than 550 South African flies to at least 320-fold coverage, we determined the genome wide TE insertion frequencies in both species. We suggest that the predominance of low frequency insertions in the two species (>80% of the insertions have a frequency <0.2) is probably due to a high activity of more than 58 families in both species. We provide evidence for 50% of the TE families having temporally heterogenous transposition rates with different TE families being affected in the two species. While in *D. melanogaster* retrotransposons were more active, DNA transposons showed higher activity levels in *D. simulans*. Moreover, we suggest that LTR insertions are mostly of recent origin in both species, while DNA and non-LTR insertions are older and more frequently vertically transmitted since the split of *D. melanogaster* and *D. simulans*. We propose that the high TE activity is of recent origin in both species and a consequence of the demographic history, with habitat expansion triggering a period of rapid evolution.

## Author Summary

Transposable elements (TE) are stretches of DNA that propagate autonomously within genomes, but it is not clear whether TEs are moving at a constant rate or if TE activity is variable. Determining the genome-wide TE content of two closely related *Drosophila* species, we show that transposition rate heterogeneity is abundant. Since TE insertions are frequently associated with a selective advantage, we suggest that the observed high TE activity may have served a central role facilitating the adaptation of the two species to their novel environments after the recent out of Africa habitat expansion.

## Introduction

Transposable elements (TE) are stretches of DNA that selfishly spread within genomes. Without any force counteracting their spread, TE numbers would exponentially grow within hosts until the accumulated TE burden causes extinction of host populations. Two mechanism have been proposed that could lead to a stable equilibrium of TE copy numbers within hosts, at which the number of insertions gained by transposition equals the number of TEs lost by purifying selection [1]. Either the effective transposition rate (i.e. number of new insertions less the number of excised TEs) may be a decreasing function of TE copy numbers or the strength of negative selection against TE insertions may be increasing with TE copy numbers [1]. One important outcome of strong negative selection is that most TE insertions in *D. melanogaster* are segregating at low population frequencies (transposition-selection balance model) [2, 3, 4]. Alternatively, TE families in *D. melanogaster* may not yet have attained a stable equilibrium. In this case, the predominance of low frequency insertions is thought to be due to recent activity (transposition burst model) [3, 5, 6]. In particular, families that recently invaded a novel host, like the P-element, may not yet have reached an equilibrium state [6, 7]. Nevertheless, given sufficient time all TE families are expected to eventually attain an equilibrium between the gain of new insertions by transposition and elimination of insertions facilitated by negative selection. The dynamics of TEs after reaching this equilibrium are not well understood. One possible outcome is that the equilibrium is stable, which results in vertical transmission as frequently seen for non-LTR transposons [8, 9]. Alternatively, the evolution of host factors [10, 11] could modulate transposition rates over time. Such fluctuations in TE activity could result in vertical extinction, especially if transposition rates reach low levels. Alternatively, a gradual and irreversible accumulation of deleterious mutations may inevitably lead to vertical extinction of some TE lineages [12, 13]. Horizontal transmission (HT) of active copies to a novel host may be a necessary step to ensure long-term maintainence of these lineages [14, 12]. While all these processes have been inferred from the analysis of TEs in extant populations, it is clear that the long-term evolution of TEs can only be understood if intraspecific TE dynamics can be connected between species that are sufficiently diverged to recognize differences, but also sufficiently close to make informative comparisons. We investigated the TE content in natural *D. melanogaster* and *D. simulans* populations, two closely related species which diverged about 2–3 million years ago [15, 16]. Using empirical TE insertion frequency estimates from Pool-Seq we show that, like in *D. melanogaster* (*f* ≤ 0.2; 87%), most TE insertions in *D. simulans* segregate at low frequencies (*f* ≤ 0.2; 80%). We propose that this is likely due to a high activity of more than 58 TE families in both species. This high TE activity may be of recent origin in both species, triggered by habitat expansion. Interestingly, retrotransposon families were more active in *D. melanogaster* while DNA transposons were more active in *D. simulans*.

## Results

We compared the TE abundance in natural populations of the two closely related species *D. melanogaster* and *D. simulans* to determine the patterns of long-term transposon activity. The comparison of TE abundance in the two species has been complicated by markedly different qualities of the reference genomes and the associated TE annotations. To avoid bias that might arise from using genomes assemblies of different quality, we pursued the following strategies: (i) using an improved *D. simulans* reference assembly [17], (ii) restricting the TE abundance comparison to orthologous regions, i.e. regions present in the assemblies of both species (iii) using the same *de novo* TE annotation pipeline in both species [annotating TEs in all currently available *D. simulans* assemblies [18, 19, 17]; see Material and Methods] and (iv) employing a TE calling method that is independent of the presence of a TE insertion in the reference genome. Our pipeline also takes sequence variation between insertions of TE families into account by mapping reads to the consensus TE sequences as well as to all sequence variations of a TE family found in the reference genome(s). From each species we analyzed isofemale lines collected 2013 in Kanonkop (South Africa). By sequencing pooled individuals (Pool-Seq) [20] we obtained an average coverage of at least 320-fold using Illumina paired end reads, which corresponds to an average physical coverage of 145 at TE insertion sites. We estimated TE abundance using PoPoolationTE [5]. The impact of the various steps in our pipeline is detailed for every TE family in S1 Table.

### Validation of our pipeline for estimating TE abundance

A comparison of *de novo* annotated TEs in *D. melanogaster* with the reference annotation [FlyBase; v5.53; [21, 22]], indicated that our pipeline for annotating TEs has a high sensitivity as well as a high specificity (S1 Text). The high quality of our TE annotation is further supported by the very similar sets of TE insertions identified in a *D. melanogaster* population [5] using our pipeline and either the *de novo* annotation of TE insertions or the reference annotation (77–91% overlap; S1 Text). Moreover the population frequency estimates and number of TE insertions in the South African *D. melanogaster* population were highly similar to the ones in a European population [5] despite that the latter one was based on the reference TE annotation (population frequency estimates: Spearman’s rank correlation, *r*_*S*_ = 0.82, *p* < 2.2*e* − 16, insertion numbers: Spearman’s rank correlation, *r*_*S*_ = 0.81, *p* < 2.2*e* − 16; S1 Text). As final validation of our annotation pipeline we compared the genomic TE distribution in natural populations obtained from our pipeline to an independently acquired data set. Vieira *et al*. (1999) estimated the abundance of 36 TE families in *D. melanogaster* and *D. simulans* populations by *in situ* hybridization. We obtained a reasonable correlation between the estimates of both methods (*D. melanogaster*: Spearman’s rank correlation, *r*_*S*_ = 0.85, *p* = 3.6*e* − 9; *D. simulans*: *r*_*S*_ = 0.62, *p* = 0.0002; S1 Text), confirming the robustness of our method. In agreement with these indicators of reliable TE identification, recent computer simulations indicated that the software used for estimating TE abundance (PoPoolationTE) has a high sensitivity [23] and TE insertions identified with this software were validated with PCR [24].

### TE abundance in a natural population of *D. simulans* and *D. melanogaster*

**Fig 1:**
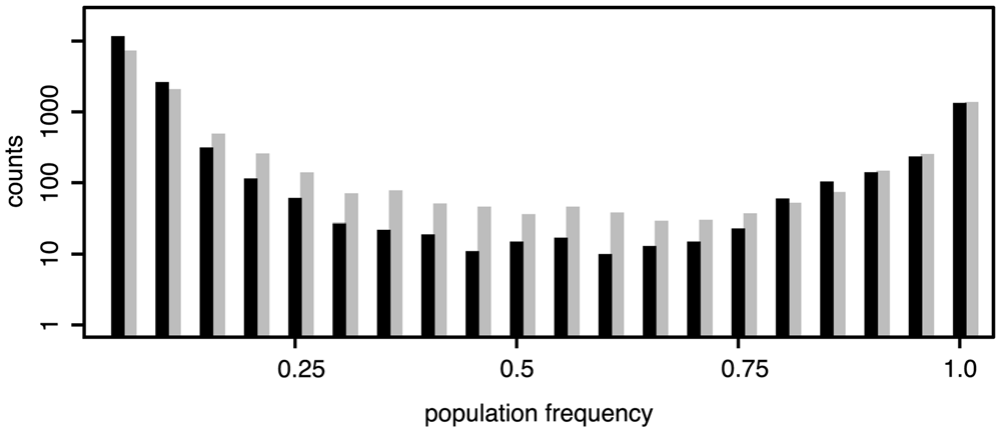
Frequency distributions of TE insertions in *D. melanogaster* (black) and *D. simulans* (grey); Only TE insertions for which the population frequencies could be estimated are shown (not overlapping, minimum physical coverage of 30); *D. melanogaster*: 16,901 insertions; *D. simulans*: 12,716 insertions.

**Fig 2:**
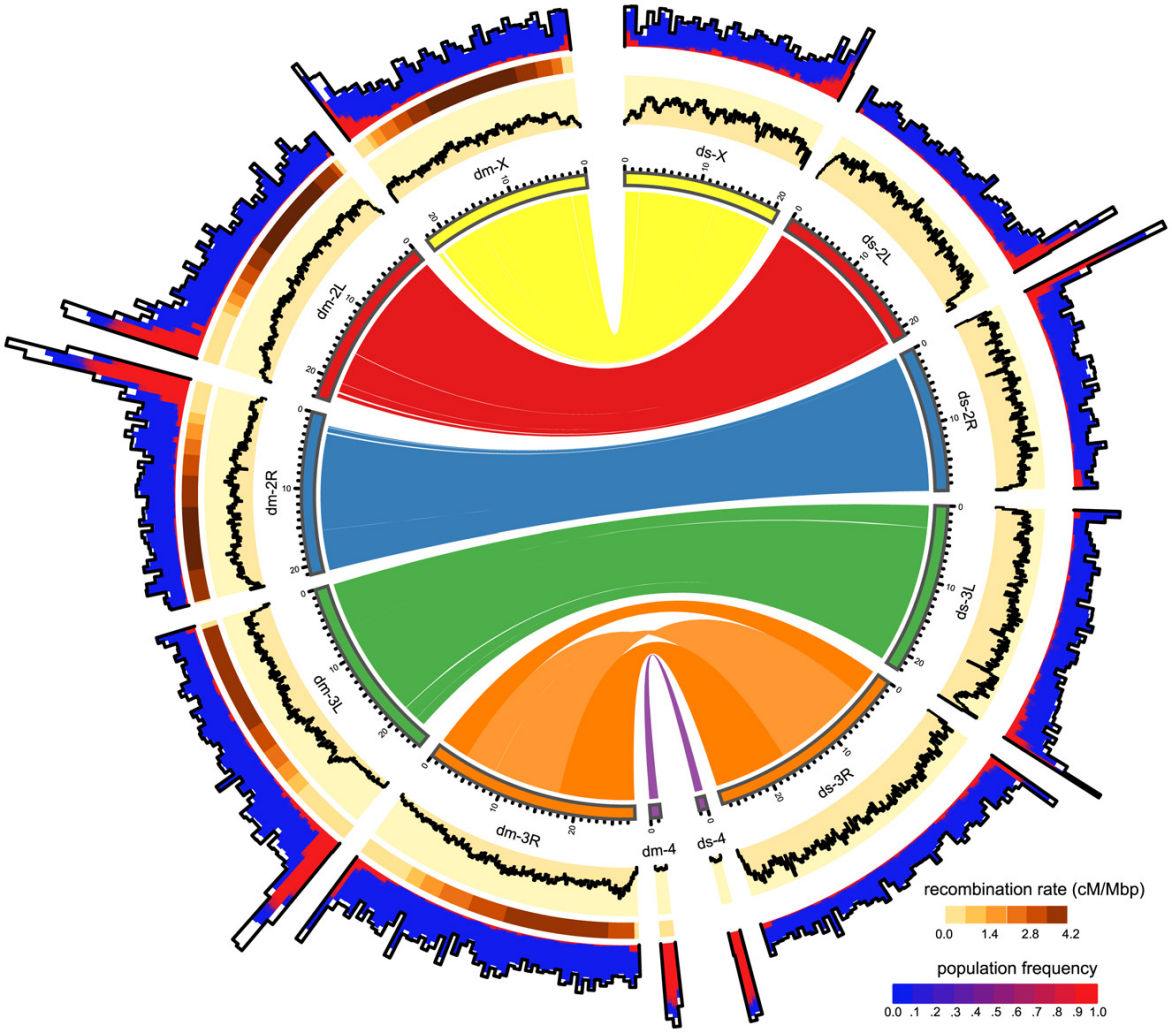
Distribution of TE insertions in a natural population of *D. melanogaster* (dm) and of *D. simulans* (ds). The TE distribution (outer graph) is compared to the recombination rate (middle graph) and the nucleotide polymorphism (Θ_*π*_, yellow inner graph). TE abundance and recombination rate are shown for windows of 500kb, whereas the nucleotide diversity is shown for windows of 100kb. For overlapping TE insertions (white) no estimates of population frequencies could be obtained. The relationship between the reference genomes is shown in the inside. Note, the inversion on chromosome 3R [47] and the missing pericentromeric regions in the assembly of *D. simulans*. The maximum nucleotide diversity of the plot is 0.018 and the maximum number of TE insertions 400.

**Fig 3:**
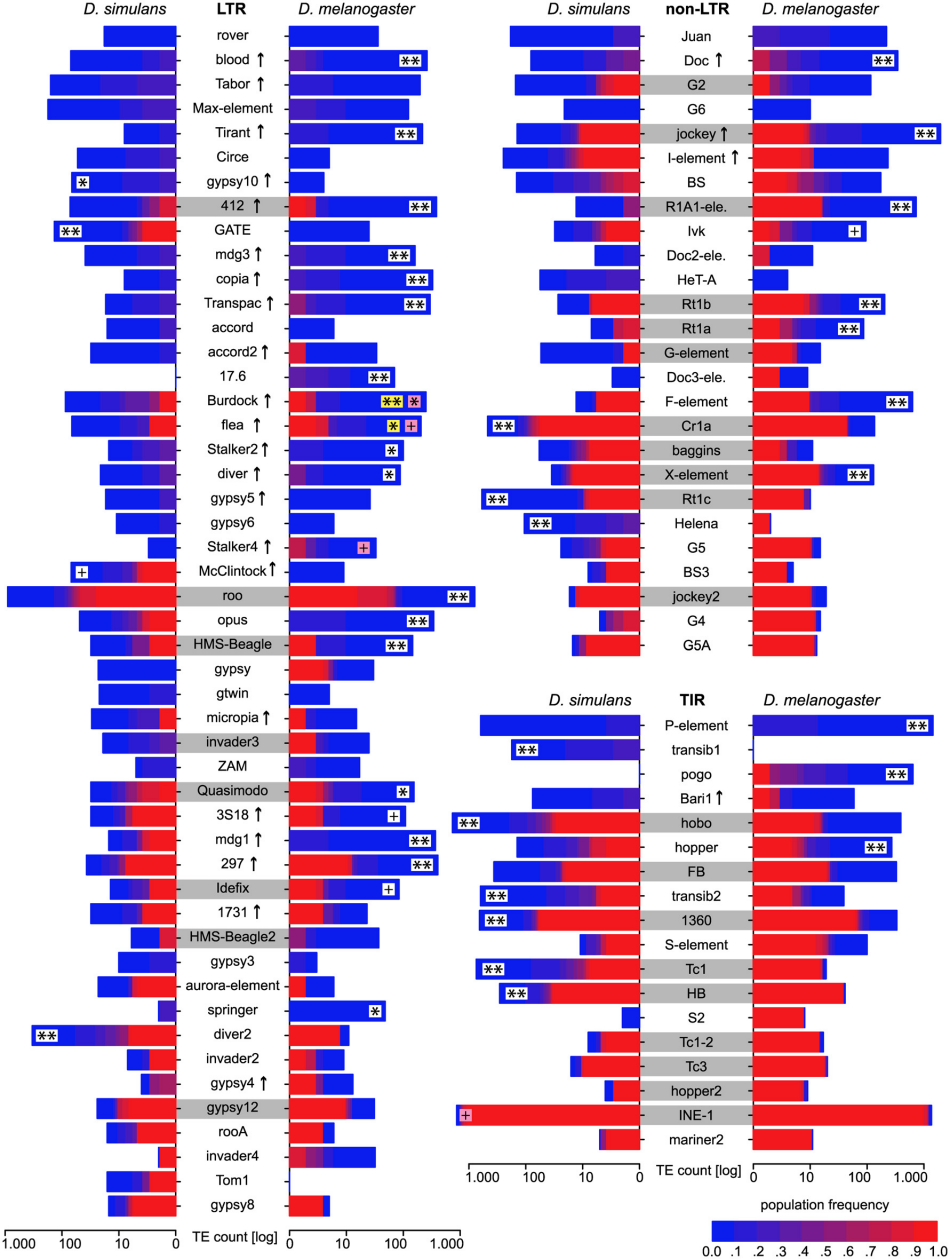
Abundance of different TE families in natural *D. melanogaster* and *D. simulans* populations; Significant differences in TE copy numbers from expectations under drift are indicated for the species with a higher number of insertions, assuming equal population sizes in both species (yellow), or a *N*_*e*_ ratio of 1.519 (pink). Those cases for which both models agree are shown in white. Families with at least one fixed insertion common to both species are highlighted in grey and families with documented HT between *D. simulans* and *D. melanogaster* [46] are marked with an arrow. p-value after Bonferroni correction: ** < 0.001; * < 0.01; + < 0.05; Only TE families having in total more than 10 insertions are shown. Foldback (FB) is grouped with TIRs solely for graphic reason.

The number of TE insertions differs markedly between the two species (Fig 2) with a larger number of TE insertions in a *D. melanogaster* population than in a *D. simulans* population (*Dmel* = 18,382, *Dsim* = 13,754, Chi-squared test, *χ* ^2^ = 666.5, *p* < 2.2*e* − 16; physical coverage = 145; minimum count = 3; orthologous regions). Analyzing only TE insertions for which population frequencies could be estimated (S2 Table) and excluding INE-1, an old and abundant TE family [25, 26], we found that this observation also holds when comparing the average number of TE insertions per haploid genome (*Dmel* = 1,275, *Dsim* = 1,172, Chi-squared test, *χ* ^2^ = 4.3, *p* < 0.037; including INE-1: *Dmel* = 2,459, *Dsim* = 2,531, *χ* ^2^ = 1.04, *p* < 0.31). A lower number of TE insertions in *D. simulans* than in *D. melanogaster* has been reported previously using *in situ* hybridization [27, 28, 29]. We found that the number of fixed insertions (*f* ≥ 0.9, allowing for some error) is very similar between the two species (*Dmel* = 1,574, *Dsim* = 1,639, Chi-squared test, *χ* ^2^ = 1.315, *p* = 0.215) and that the different TE abundance between populations of the two species is mostly due to low frequency insertions (*f* ≤ 0.2, *Dmel* = 14,789, *Dsim* = 10,203, Chi-squared test, *χ* ^2^ = 841.5, *p* < 2.2*e* − 16). We confirm the previously reported predominance of low frequency insertion in *D. melanogaster* [30, 31, 2, 5, 32] and show that the same pattern, albeit to a slightly lesser extent (*f* ≤ 0.2, *Dmel* = 87.5%, *Dsim* = 80.2%; Fig 1) is present in *D. simulans*. In agreement with this, the average population frequency of TE insertions is higher in *D. simulans* (0.199) than in *D. melanogaster* (0.146). As heterochromatic regions may contain substantial fractions of TE insertions (S1 Table) and the two reference genomes include different amounts of heterochromatin, the absence of insertions of a TE family in a comparison of orthologous regions (Fig 2), does not necessarily imply that this family is truly absent. Despite these limitations, we do not find species specific TE families. All 121 investigated TE families are present in both *D. simulans* and *D. melanogaster* (with the exception of Stalker3, which may be missing in *D. simulans*; S1 Table). Analyzing the different TE classes separately, we uncovered pronounced differences in TE abundance between the two species. *D. melanogaster* (i.e. the *D. melanogaster* population from South Africa) has markedly more Long Terminal Repeat (LTR; *Dmel* = 7,252, *Dsim* = 3,222; Fisher’s Exact Test *p* < 2.2*e* − 16) and non-LTR (*Dmel* = 5,723, *Dsim* = 2,902; Fisher’s exact test *p* < 2.2*e* − 16) insertions, whereas *D. simulans* has more Terminal Inverted Repeat (TIR) insertions (*Dmel* = 5,021, *Dsim* = 7,258; Fisher’s exact test *p* < 2.2*e* − 16). Many RNA transposon families (LTR and non-LTR) have more insertions in *D. melanogaster* whereas DNA transposon families (TIR) are more abundant in *D. simulans* (Fig 3). The unexpected presence of the P-element in *D. simulans* [Fig 3; [33, 34, 29]] is discussed elsewhere [24]. Despite these differences, the TE abundance is very similar between *D. melanogaster* and *D. simulans* (Spearman’s rank correlation of TE copy numbers for every family; *r*_*S*_ = 0.57, *p* = 2.3*e* − 11; Fig 3). The similarity is higher for fixed TE insertions (Spearman’s rank correlation of fixed, *f* ≥ 0.9, insertions; *r*_*S*_ = 0.73, *p* < 2.2*e* − 16) than for low frequency insertions (Spearman’s rank correlation of low frequency, *f* ≤ 0.2, insertions; *r*_*S*_ = 0.52, *p* = 7.5*e* − 10). This high similarity of the abundance of fixed insertions is not unexpected as fixed insertions are highly enriched for insertions shared between *D. melanogaster* and *D. simulans* (Fisher’s exact test; *p* < 2.2*e* − 16; S2 Text), which likely predate the split between these two species about 2–3 million years ago [15, 16].

### Temporal heterogeneity of transposition rates

**Fig 4:**
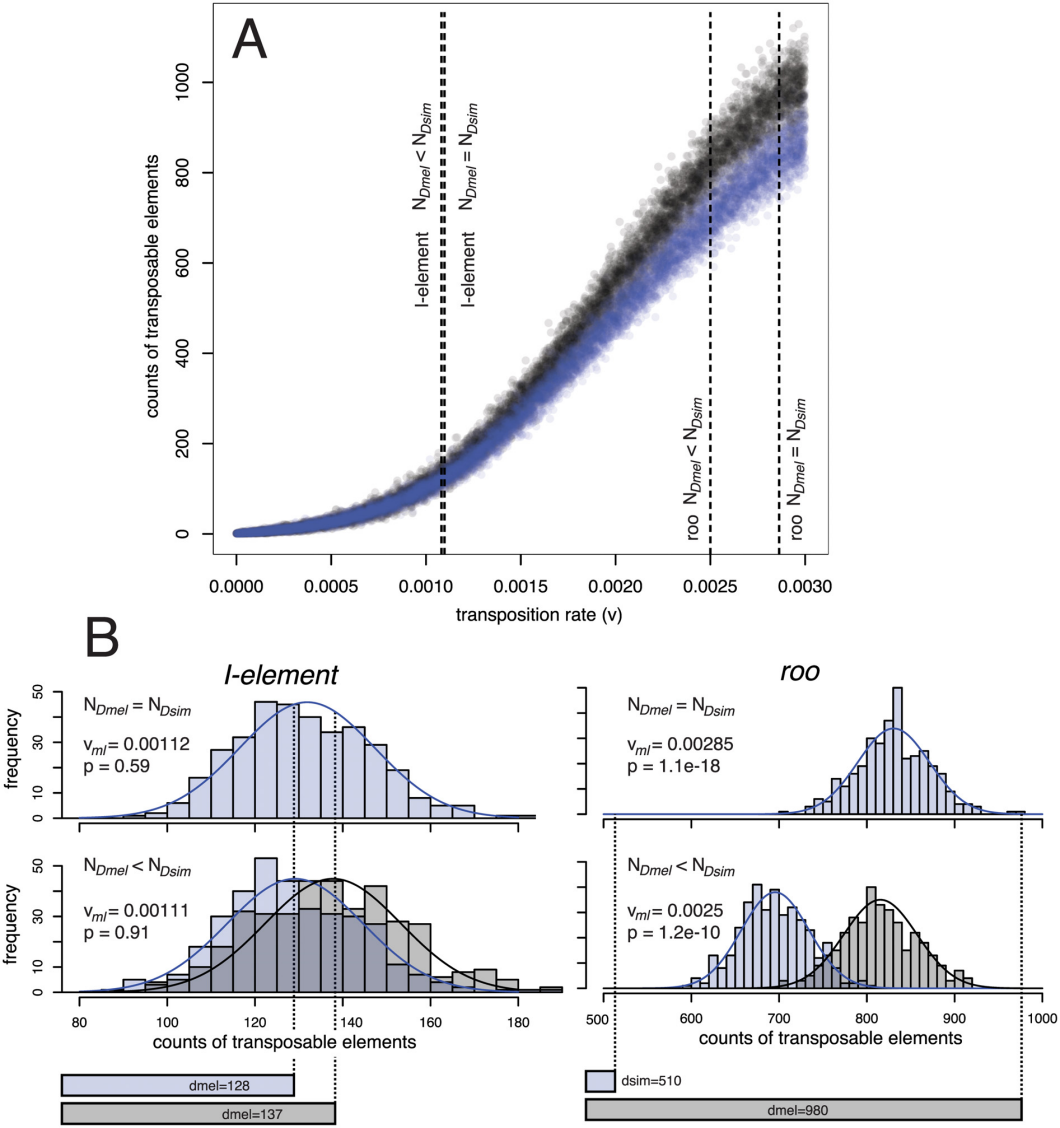
Procedure for estimating the significance (*p*) of the difference in TE copy numbers between *D. simulans* (Dsim) and *D. melanogaster* (Dmel) from expectations under drift using an equilibrium model. A.) Simulated equilibrium copy numbers of TE insertions for transposition rates (*v*) ranging from 0 to 0.003 and two different populations sizes (*N* = 6,583 black dots; *N* = 10,000 blue dots). More than 10.000 independent simulations were performed for each population size. For every TE family (e.g. *roo* and *I-element*) the maximum likelihood transposition rate (*v*_*ml*_) is identified, assuming either an about 1.519 times smaller population size in *D. melanogaster* than in *D. simulans* (*N*_*Dmel*_ < *N*_*Dsim*_) or equal population sizes in both species (*N*_*Dmel*_ = *N*_*Dsim*_). B.) A normal distribution is fitted to the equilibrium copy numbers in a small window around *v*_*ml*_ and *p* can be estimated from the two tailed area obtained by intersecting the normal distributions with the observed copy numbers in the two species (bottom bars). For details see material and methods.

To test if the observed differences in the TE abundance between the two species could be caused by heterogenous transposition rates, we performed computer simulations. For each TE family we tested whether the observed interspecific differences in copy number (Fig 3) deviate significantly from expectations under drift using an equilibrium model in which we assume that the transposition rate and the selective effects are the same in both species. Our simulations considered each TE family separately and relied on a fitness function in which fitness decreases exponentially with insertion numbers, a necessary condition for obtaining stable equilibria [1]: $w_{i}=1-xg_{i}^{t}$, where *w*_*i*_ is the fitness of a given individual, *x* the selective impact of a TE insertions, *g*_*i*_ the number of TE insertions found in a given individual and *t* the degree of synergism between TE insertions (needs to be > 1.0 for stable equilibria). We refrained from simulating other models that would also lead to stable equilibria, which either require that the transposition rate decreases or that the excision rate increases with insertion numbers [1], as there is little support for these models [10]. Given the strong influence of population size on TE dynamics [35, 36] (S3 Text), we used a population size ratio in our computer simulations that reflects the ratio of the population variation estimator *π* (*π* ^*Dsim*^/*π* ^*Dmel*^ = 0.0113/0.0074 = 1.519; S4 Text). These simulations provide the probability (*p*) that the observed difference in TE copy numbers between *D. simulans* and *D. melanogaster* is compatible with the null hypothesis of an equilibrium model with genetic drift, constant transposition rates, and equal negative selection against TE insertions. In about 50% (46/93) of the TE families the number of insertions deviated significantly from expectations under drift after accounting for differences in population size (Fig 3; see Fig 4 for an illustration of the procedure used for identifying significant deviations). This result was robust with respect to a wide range of different population sizes (*N* ≥ 10,000; S3 Text). Also when assuming an equal population size of the two species (e.g. [37]) substantial deviations from expectations under drift were identified (Fig 3). Furthermore, our results are robust to recombination rates allowing even higher ones than those reported for *D. melanogaster* [as may be found in *D. simulans* [38]] as well as over a wide range of other parameters (*t* ≥ 1.3, *x* ≥ 0.0004; S3 Text). Relaxing these parameters further (e.g. *t* < 1.3) quickly results in conditions under which purifying selection against TEs is too weak to maintain stable TE copy numbers, leading to extinction of the host population (S3 Text). The fraction of families with heterogenous transposition rates is roughly similar for all three TE orders [LTR 51% (25/49), non-LTR 42% (11/26), TIR 55% (10/18)]. RNA transposons (LTR and non-LTR) are significantly more active in *D. melanogaster* while DNA transposons (TIR) are more active in *D. simulans* (Dsim *RNA* = 7, *DNA* = 7; Dmel *RNA* = 29, *DNA* = 3; Fisher’s exact test; *p* = 0.0045). It is important to note that these results are based on the assumption that TE families evolve in transposition-selection balance [3] which, although probably true for most TE families [7], may not hold for families that recently invaded a novel host, like the P-element [7]. Especially LTR transposons could be of very recent origin and thus not yet in transposition-selection equilibrium [6, 5]. Therefore, we separately analysed TE families that are likely vertically transmitted as a conservative set of TE families in transposition-selection balance. We identified families with at least one shared TE insertion between *D. simulans* and *D. melanogaster* [only high frequency insertions, *f* ≥ 0.8, were considered as the strong insertion bias of some TE families may lead to shared low frequency insertions [24]; Fig 3], suggesting vertical transmission since the split of the two species. In total 28 families had at least one shared insertion, with TIRs having the most and LTRs the least [TIR 50% (9/18), non-LTR 42% (11/26), LTR 16% (8/49)]. For about 57% (16/28) of vertically transmitted families the TE abundance between the two species significantly deviated from expectations under drift (Fig 3).

### Intraspecific heterogeneity of transposition rates in *D. melanogaster*

The large number of species specific TE activity patterns encouraged us to evaluate the distribution of TEs between two *D. melanogaster* populations from South Africa and Portugal. We observed substantial differences in TE abundance for two families (R1A1-element, gypsy2; S1 Text). This pattern is in agreement with previous observations [39, 29] suggesting that the TE composition of local Drosophila populations can differ markedly despite little differentiation among cosmopolitan *D. melanogaster* populations [40, 41].

### Age distribution of TE insertions

The age distribution of TE insertions is an important parameter describing the dynamics of TEs. A direct approach to determine the age of TE insertions is based on the number of mutations after insertion [6, 9, 42, 43], but this method cannot be applied to Pool-Seq data. Nevertheless, the previously demonstrated strong correlation between sequence divergence of TEs and their frequency in a natural *D. melanogaster* population [5] suggests that population frequencies of TE insertions are good age estimators, with young insertions mostly segregating at low population frequencies while old insertions frequently have higher population frequencies. We further scrutinized this relationship by reasoning that young TE insertions are more likely to be expressed. Using RNA-Seq data from *D. simulans* [17] we found a significant negative correlation (Spearman’s rank correlation, *r*_*S*_ = −0.34, *p* = 0.00024;S1 Fig) between population frequency and expression intensity. By contrast, we show that fixed TE insertions are mostly old as we found them to be enriched for insertions predating the split between *D. simulans* and *D. melanogaster* (see above and S2 Text). Overall, our analyses suggested that the population frequency of TE insertions provides a rough, but suitable estimator for the age of TE insertions. Based on this estimator we suggest that low frequency insertions are mostly due to recent TE activity. Hence, the predominance of low frequency insertions in *D. melanogaster* and *D. simulans* is due to recent activity of multiple TE families in both species (*f* ≤ 0.2, *Dmel* = 87.5%, *Dsim* = 80.2%), where 58 families (62%; 58/93) in *D. melanogaster* and 64 (68%; 64/93) families in *D. simulans* have more than 10 low frequency insertions. The five families with the lowest population frequency, and thus likely the most recently active TE families, in *D. melanogaster* are: P-element, Tirant, R2-element, copia and mdg1; and in *D. simulans*: P-element, R2-element, gypsy, G6 and accord2 (see S3 Table for full data set). This inference could be confirmed for the P-element, which invaded both species only within the last few decades [44, 24]. In both species LTR insertions have, on the average, the lowest population frequency whereas TIR insertions have the highest (Dsim *LTR* = 0.11, *non* – *LTR* = 0.13, *TIR* = 0.26; Dmel *LTR* = 0.07, *non* – *LTR* = 0.08, *TIR* = 0.33) suggesting that in both species LTR insertions are mostly of recent origin. This is in agreement with previous work which showed that LTR insertions in *D. melanogaster* are mostly young [6, 45]. We found that the average population frequency of TE families is correlated between *D. simulans* and *D. melanogaster* (Spearman’s rank correlation for families having at least one insertion in both species; *r*_*S*_ = 0.57, *p* = 5.0*e* − 10). This correlation is strongest for TIR transposons and weakest for LTR transposons (LTR *r*_*S*_ = 0.43, *p* = 0.001; non-LTR *r*_*S*_ = 0.59, *p* = 0.0004; TIR *r*_*S*_ = 0.81, *p* = 7.0*e* − 05), which suggests that the timing of activity is most similar between TIR families and the least between LTR families. We propose that this could be the outcome of different modes of transmission of TEs. Previous studies suggested that non-LTR transposons may be preferentially vertical transmitted [8, 9]. In agreement with this we found a high fraction of vertically transmitted TE families (estimated as families sharing one high frequency insertion between the two species; see above) for non-LTR but also for TIR transposons. LTR transposons had the smallest fraction of vertically transmitted families [*LTR* = 16% (8/49), *non* – *LTR* = 42% (11/26), *TIR* = 50% (9/18); Fig 3]. Conversely, in a scan for evidence of horizontal transfer of TEs between *D. simulans* and *D. melanogaster*, Bartolome *et al*. [46] found putative HT for many LTR families but only for a few non-LTR and TIR families [S1 Table from [46]; *K*_*s*_ < 0.04; *LTR* = 81% (26/32), *non* – *LTR* = 23% (3/13), *TIR* = 33% (1/3); Fig 3]. It is thus possible that vertical transmission is more frequent for TIR and non-LTR transposons, while HT is more frequent for LTR transposon. This could account for the weak correlation of average age of TE insertions (as measured by population frequency) of LTR families and the strong correlation of non-LTR and TIR families, as vertical transmission may result in more predictable temporal development of TE activity than HT, which is a highly stochastic process (e.g. [24]).

## Discussion

In this report, we provide the first genome-wide characterization of TE abundance in large population samples of the two closely related species *D. simulans* and *D. melanogaster*. Consistent with previous reports [29, 48], we found considerable differences in TE composition between the two species.

We show that in both species, *D. simulans* and *D. melanogaster*, most TE insertions segregate at of low population frequencies. We propose that this predominance of low frequency insertions is most likely due to a high activity of multiple (> 58) TE families in both species, which raises the important question whether this high activity is continuously maintained, e.g. since the split of the two species, or is of recent origin. Based on the observation that TE abundance in ancestral African populations of *D. melanogaster* is lower than in populations of other continents and because of the generally high heterogeneity of TE abundance in *D. simulans* populations, Vieira *et al*. [29] suggested that the recent habitat expansion of *D. simulans* and *D. melanogaster* may have triggered bursts of TE activity in these two species [49, 50]. Colonization of new environments may trigger increased TE activity by two, not mutually exclusive mechanisms: either stress associated with new environments disturbs systems that guard against TE proliferation, such as piRNA, or the habitat expansion may bring species into contact, that not co-existed previously. In combination with horizontal transfer of TEs, this could result in activity of a TE in a new host [34, 51]. One classic example for this scenario is the transfer of the P-element from *D. willistoni* to *D. melanogaster*, which invaded the territory of *D. willistoni* in South America [34]. After the horizontal transfer, the P-element rapidly spread in *D. melanogaster* populations worldwide [52]. Moreover, previously dormant TE families may also become reactivated upon the activation of a single TE family, as has been noted during hybrid dysgenesis [53, 54], where DNA damage mediated stress seems to be causative [54, 55, 53].

### TE activity increased recently

The hypothesis of a recent increase in TE activity in both *D. melanogaster* and *D. simulans* is supported by several lines of evidence. First, based on computer simulations we find transposition rate heterogeneity in 50% (46/93) of TE families. Since our test is designed to detect differences between the two species and at least some TE families have recently increased their transposition activity in both species it is likely that the phenomenon of transposition rate heterogenetiy is even more common than our data suggests. For example, the P-element has a high, albeit unequal, activity in both *D. simulans* and *D. melanogaster*, but it only invaded both species within the last 100 years [44, 24]. Another example is the I-element, which has about equal activity in both species, but it was suggested that active copies of the I-element were lost in *D. melanogaster* some time ago, and that active copies only recently reinvaded extant populations [56] (Fig 3). Furthermore, differences in TE composition are not only recognized in between-species comparisons, but can be also detected between two *D. melanogaster* populations (S1 Text). These differences are unlikely to result only from demographic events since these should affect all TE families equally, whereas we only found marked differences for two TE families. Such differences in TE abundance between populations have also been observed in *D. simulans* [39]. Third, LTR transposons may be of recent origin in *D. melanogaster* [6, 45]. Based on low population frequencies we suggest that this probably also holds for LTR insertions in *D. simulans*. Consequently, LTR insertions may be of very recent origin in both species. Fourth, HT of TEs, one mechanism by which habitat expansion could trigger bursts of TE activity, has been reported to be abundant in *D. melanogaster* especially for LTR transposons [46, 57].

In summary, we conclude that the TE composition in *D. simulans* and *D. melanogaster* is probably dynamic and changes quickly, such that inter-population differences can also be detected. It is therefore conceivable that the high TE activity in *D. melanogaster* as well as in *D. simulans* is of recent origin. With TE insertions frequently contributing to adaptation to novel environments [58, 5], increased transposition rates may be an important component of successful habitat expansions.

### Uncertainty about TE features affect the generality of computer simulations

Since it is well understood that the distribution of TE insertions is strongly affected by population size [1, 59], any comparison of TEs in two closely related species needs to account for heterogeneity in genetic drift due to different population sizes in both species. Our computer simulations suggest that the observed differences in copy numbers could not be explained by genetic drift for about half of the TE families. Nevertheless, differences in TE abundance may either be due to differences in transposition rates or strength in purifying selection removing TE insertions. Since population size [59] and the recombination rate [60], the major factors modulating the strength of selection against TE insertions, affect all families similarly, our data are not compatible with unequal purifying selection. The observation that some families are more abundant in *D. simulans* while other families are more abundant in *D. melanogaster* strongly suggests the presence of family specific factors that evolved heterogeneously in the different lineages. As family specific divergence of transposition rates has also been documented previously [11, 61, 53, 54], we propose that heterogenous transposition rates are the most likely explanation for significant differences in TE abundance between the two species. However, our computer simulations made several assumptions about the behaviour of TEs and, like for all models, the conclusions drawn are strongly dependent on the parameters used in the computer simulations. Unfortunately, very little is known about the key parameters determining the dynamics of TEs: i) Which of the three equilibrium models (decreasing fitness, decreasing transposition rate, increasing excision rate) or which combination of these three models [1] reflects reality best? ii) Which fitness function most accurately describes the relationship between TE copy number and fitness? iii) What are the biological realistic values of the parameters entering the fitness functions? iv) Is a model assuming co-dominant, recessive or dominant effects of TE insertions closest to reality? v) What are the exact recombination rates of *D. melanogaster* and *D. simulans*? vi) Should differences in recombination rates enter the fitness function and if so which function best describes this effect (for example, due to the deleterious effects of ectopic recombination, it is possible that the selective impact of a given TE insertion depends on the recombination neighborhood)? vii) Are more complex demographic scenarios necessary—for example those involving migration—and if so which is the exact demographic history of the two populations? Since it is not possible to consider all these factors in our computer simulations we decided to rely on commonly used default parameters [co-dominant model with exponentially decreasing fitness function; *t* = 1.3; *x* = 0.0004; 0.0 ≥ *v* ≥ 0.003 (e.g. [1, 62]) and to closely reproduce the genomic landscape of *D. melanogaster* [68,700,000 million insertion sites in high recombining regions (> 1cM/Mbp) on four chromosome arms; the recombination rate of *D. melanogaster* [63]]. Finally, our simulations reproduce the sampling properties of our study (145 haploid genomes with a minimum count per TE insertion of 3).

### Does the TE composition reflect a different colonization history?

Interestingly, retrotransposon families are more active in *D. melanogaster* while DNA transposons are more active in *D. simulans*. This contrast may be the outcome of different propensities for horizontal transfer among the major TE groups (LTR, non-LTR, TIR) in combination with the different colonization times of *D. melanogaster* and *D. simulans*. DNA transposons (TIR) and LTR transposons seem to be more prone to horizontal transfer than non-LTR TEs, since their double stranded DNA intermediates may be more stable than the RNA intermediate of non-LTR TEs [64, 9]. Furthermore, the integration of DNA transposons requires only transposase and no specific host factor, which makes these TEs potentially more successful invaders of diverged genomes [64, 51]. The very recent out of Africa habitat expansion of *D. simulans* [65] about 100 years ago is therefore consistent with the higher activity of DNA transposons. *D. melanogaster*, on the other hand, colonized Europe already more than 10,000 years ago [66], providing sufficient time for less invasive retrotransposons to colonize a new host. Furthermore, if *D. melanogaster* experienced a burst of DNA TEs shortly after the colonization, the host defense system (e.g.: the piRNA system [67]) may have matured to control the initially invading DNA TEs. Under this scenario, the genomic TE signature in *D. simulans* is expected to experience a transition from high activity of DNA transposons to high activity of retrotransposon in the next couple of centuries. However, a high propensity for HT of TIR transposons [64, 51] could be interpreted to counter our observation that many TIR families are vertically transmitted. Nevertheless, TEs like the I-element may invade hosts in multiple waves [56], and HT could therefore be abundant even for vertically transmitted TE families. Families with evidence for both vertical and horizontal transmission, like 412 and jockey (Fig 3), may have experienced multiple waves of invasion.

The likely role of habitat expansions for TE activity raise questions regarding genomic TE distributions in species that remained in their original habitat. Does this imply that TE activity is lower in endemic species? The analysis of ancestral African *D. melanogaster* and *D. simulans* populations may help to resolve this question as well as related *Drosophila* species that remained in their ancestral habitat. Furthermore, monitoring TE abundance in experimentally evolving populations may shed some light on the dynamics of TEs in populations and on the short term evolution of transposition rates. Finally, long read sequencing could provide a better characterization of TE insertions [68], which may help unraveling the phylogenetic relationship of TEs and thus provide some clues on the role of vertical and horizontal transmission in the life-cycle of TEs.

## Materials and Methods

### Fly samples and sequencing

We collected 1,300 isofemale lines of *D. simulans* and 1,250 isofemale lines of *D. melanogaster* from Kanonkop (South Africa) in 2013. The lines were kept in the laboratory for 8 generations. We used a single female from 793 (554) isofemale *D. simulans* (*D. melanogaster*) lines for pooling. Genomic DNA was extracted from pooled flies using a high salt extraction protocol [69] and sheared using a Covaris S2 device (Covaris, Inc. Woburn, MA, USA).

We used three different protocols to prepare paired-end libraries. One library (BGI-91a; S4 Table) was prepared following a modified version of the NEBNext Ultra protocol (New England Biolabs, Ipswich, MA). For another library (BGI-92a, BGI-92b, BGI-93b; S4 Table) we used the NEXTflex PCR-Free DNA Sequencing Kit (Bioo Scientific, Austin, Texas) with modifications. The third library (BGI-93a; S4 Table) was prepared based on the NEBNext DNA Sample Prep modules (New England Biolabs, Ipswich, MA) in combination with index adapters from the TruSeq v2 DNA Sample Prep Kit (Illumina, San Diego, CA). All protocols made use of barcoding (S4 Table). For each library we selected for a narrow insert size, ranging from 260–340, using agarose gels. A total of five lanes 2x100bp paired-end reads were sequenced on a HiSeq2000 (Illumina, San Diego, CA). In summary we sequenced 364 million paired end fragments for *D. melanogaster* and 288 million paired end fragments for *D. simulans* (S5 and S6 Tables). This yields an average coverage of 381 in *D. melanogaster* and of 327 in *D. simulans*.

### Annotation of TE insertions

One of the requirements for estimating the abundance of TE insertions with PoPoolation TE [5] is a reliable TE data base. A manually curated high-quality annotation of TE insertions has been generated for *D. melanogaster* [22, 21], whereas, to our knowledge, so far no TE annotation of comparable quality exists for *D. simulans*. To avoid any biases that may result from using TE annotations of different qualities we decided to *de novo* annotate TE insertions in both species with an identical pipeline. The reference sequence of *D. melanogaster* (v5.53) was obtained from FlyBase (http://flybase.org). We used the reference sequence published by Palmieri *et al*. [17] for *D. simulans*, as this assembly is of a higher quality than the previously available one [18] and of similar quality as a recently published one [19]. We also obtained a library containing the consensus sequences of *Drosophila* TEs (transposon_sequence_set.embl; v9.42; [21]) from FlyBase. To avoid identification of spurious TE insertions we excluded canonical TE sequences not derived from *D. melanogaster* or *D. simulans* (Casey Bergman; personal communication). We mapped the consensus TE sequences against both reference genomes with RepeatMasker open-4.0.3 [70] using the RMBlast (v2.2.28) search engine and the settings recommended by [71] (-gccalc -s -cutoff 200 -no_is -nolow -norna -gff -u), yielding a raw annotation of TE insertions. The consensus sequences of several TE families contain microsatellites which may, as an artefact, be annotated as TE insertions [71, 21]. To account for this, we identified microsatellites in both reference genomes with SciRoKo 3.4 [72] (required score 12; mismatch penalty 2; seed length 8; seed repeats 3; mismatches at once 3), converted the output into a ‘gtf’ file and removed TEs from the raw annotation that overlapped with a microsatellite over more than 30% of the length using bedtools (v2.17.0; intersectBed -a rawannotation.gff -b microsatellites.gff -v -f 0.3) [73]. Overlapping TE insertions of the same family were merged and disjoint TE insertions of the same family were linked using an algorithm that, similar to dynamic programming, maximizes the score of the linked TE insertions (*match* – *score* = 1, *mismatch* – *penalty* = 0.5). We resolved overlapping TE insertions of different families by prioritizing the longest TE insertion and iteratively truncating the overlapping regions of the next longest insertions. Finally we filtered for TE insertions having a minimum length of 100 bp.

### Estimating the abundance of TE insertions with PoPoolation TE

Estimating the abundance of TE insertions with PoPoolation TE requires paired end sequences from natural populations, a reference sequence, an annotation of TE sequences and a hierarchy of the TE sequences [5]. We extracted the hierarchy of TE sequences from the database of consensus TE sequences (v9.42; see above). We extracted the sequences of the annotated TE insertions from the reference genomes into a distinct file and subsequently masked these TE sequences within the reference genome with the character ‘N’. We than concatenated the individual fasta records of (i) the consensus sequences of TE insertions, (ii) the TE sequences extracted from the reference genome and (iii) the repeat masked reference genome into a single file, which we call TE-merged-reference. Short read mapping software usually only allows for a few mismatches between read and reference genome which may lead to underestimating the abundance of some TE insertions, especially when the TE sequences are highly diverged [5]. Such a high divergence between reads and the reference sequences may also result when the consensus sequences of TE families are derived from a different species. This could lead to underestimating the abundance of TE insertions in *D. simulans* when using consensus sequences that are mostly derived from *D. melanogaster*. Therefore, we improved the sensitivity of our pipeline for *D. simulans* by including TE sequences extracted from the assemblies of Begun *et al*. [18], Palmieri *et al*. [17] and Hu *et al*. [19] (using the same TE annotation pipeline as described above) into the TE-merged-reference of *D. simulans*.

We mapped 364 million PE fragments of *D. melanogaster* and 288 million PE fragments of *D. simulans* (see above) to the respective TE-merged-reference with bwa (v0.7.5a) [74] using the bwa-sw algorithm [75] (S5 and S6 Tables). We used ‘samro’ to restore the paired end information [5]. We estimated the abundance of TE insertions with PoPoolation TE similarly as described in [5] using the following settings: identify-te-insertions.pl –te-hierarchy-level family, –min-count 3, –min-map-qual 15, –narrow-range 100; crosslink-te-sites.pl –min-dist 85, –max-dist 300; estimate-polymorphism.pl –te-hierarchy-level family, –min-map-qual 15; Subsequently we filtered for TE insertions located on the major chromosome arms (X, 2L, 2R, 3L, 3R, 4) and for TE insertions having a minimum physical coverage of 30 (physical coverage as defined here is the sum of paired end fragments that either confirm the presence or the absence of a TE insertion). An unbiased comparison of the abundance of TE insertions between different species requires similar physical coverages in all species. We therefore iteratively subsampled paired-end fragments and repeated TE identification with PoPoolation TE, until we obtained similar physical coverages in both species (S7 Table). The full information about the effect of each step of the pipeline used for estimating TE abundance is enclosed in S1 Table. This file shows for every TE family the number of mapped reads, the number of paired-end fragments supporting a TE insertion, and the TE insertions finally identified during various filtering steps.

### Estimating nucleotide polymorphism

We estimated genome-wide levels of nucleotide diversity in the two natural populations using Pool-Seq data and PoPoolation [76]. First, we aligned all reads to the respective reference genome (unmodified) with bwa aln (0.7.5a) [74] and the following parameters: -I -m 100000 -o 1 -n 0.01 -l 200 -e 12 -d 12; Duplicate reads were removed with Picard (v1.95; http://picard.sourceforge.net/). Reads with a mapping quality lower than 20 or reads not mapped as proper pairs were removed with samtools (v0.1.19) [77]. We created a pileup file for each population with samtools (v0.1.19) [77] and the following parameters: -B -Q 0; As alignments spanning indels are frequently unreliable and may lead to spurious SNP calls we removed regions flanking indels (5bp in each direction; minimum count of indel 4) from the pileup with PoPoolation [76]. Subsequently we subsampled the pileup to a uniform coverage of 175 with PoPoolation [72] and the following parameters: –max-coverage 1400 –min-qual 20 –method withoutreplace; Finally we calculated *π* for windows of 100kb with PoPoolation and the following paramters: –min-count 4 –min-coverage 165 –max-coverage 175 –min-covered-fraction 0.6 –min-qual 20 –no-discard-deletions –pool-size 1300;

### Expression level of transposable element families in *D. simulans*

To measure the expression level of different TE families in *D. simulans* we obtained previously published RNA-seq reads [17], derived from a mix of several developmental stages of *D. simulans* strain M252. The reads were trimmed with PoPoolation v1.2.2 (trim-fastq.pl) [76] using the following parameters: –fastq-type illumina, –quality-threshold 20, –min-length 40; We mapped the RNA-seq reads to a database consisting of the repeat masked reference genome of *D. simulans* [17] and the library of TE sequences derived from all three assemblies of *D. simulans* (see above). Reads were mapped with bwa (v0.7.5a) [74] using the bwa-sw algorithm [75]. Subsequently we counted the number of reads mapping to each TE family and normalized counts by the length of the consensus sequence (transposon_sequence_set.embl; v9.42; see above).

### Orthologous regions between *D. melanogaster* and *D. simulans*

The assemblies of *D. melanogaster* and *D. simulans* are of different quality, for example varying in the amount of assembled heterochromatin. An unbiased analysis of TE abundance should therefore be restricted to genomic regions being present in the assemblies of both species. We identified these regions by aligning the genomes of *D. melanogaster* (v5.53) and *D. simulans* [17] with MUMmer (v3.23; nucmer) [78]. To avoid spurious alignments we masked all sequences derived from TEs in both reference genomes (see above) prior to the alignment. Coordinates were extracted with the ‘show-coords’ tool [78] and only alignments of the major chromosome arms (X, 2L, 2R, 3L, 3R, 4) were considered. Due to the masking of TE sequences these raw alignments contain a plenitude of gaps where the TE insertions actually causing the gaps are not found in genomic regions that are present in the alignment. To mitigate this we linked these gaps by merging alignments not separated by more than 20,000bp in both species. This threshold of 20,000bp was arbitrarily chosen because only six of the masked regions in the repeat-masked genome of *D. melanogaster* have a size larger than 20,000bp.

### Modeling TE abundance in populations under an equilibrium model

We performed forward simulations for estimating the variance of TE abundance in natural populations expected under an equilibrium model. The simulations aimed to capture conditions found in *D. melanogaster* and accordingly we (i) simulated diploid organisms, (ii) used a genome with a similar size and number of chromosomes as *D. melanogaster* and (iii) used the recombination rate of *D. melanogaster*. We obtained the recombination rate from the *D. melanogaster* recombination rate calculator v2.2 [63] for windows of 1000kb. We excluded the X-chromosome and low recombining regions (< 1cM/Mbp)- including the entire chromosome 4—from the analysis (for both the simulations and the actual data to which the simulation results are compared to). In summary we performed our simulations with *T* = 68,700,000 TE insertions sites (distributed over the following genomic regions 2L:300,000–16,600,000, 2R:3,900,000–20,700,000, 3L:900,000–17,400,000, 3R:6,600,000–25,700,000) where every insertion site may either be empty or occupied. In our model, every TE insertion has a constant probability of transposing to a novel site *v* and excision events (*u* = 0) were not considered. Novel TEs were randomly inserted in any of the *T* insertion sites at any of the two haploid genomes. If an insertion site was already occupied the transposition event was ignored. For any individual *i* in a population of size *N* the fitness *w*_*i*_ can be calculated as $w_{i}=1-xg_{i}^{t}$, where *g*_*i*_ is the number of TE insertions, *x* is the selective disadvantage of each insertion and *t* represents the interactions between the insertions [1]. This is a model where all TE insertions exert a semi-dominant effect [1].

Per default we used *x* = 0.0004 and *t* = 1.3 in our simulations. We furthermore used fecundity selection, where any individual has a probability of mating *p*_*i*_ that linearly scales with fitness *w*_*i*_ ($p_{i}=w_{j}/N\bar{w}$; $\bar{w}$ is the average fitness; after [79]).

We simulated evolving populations with non-overlapping generations, proceeding at every generation in the following order: First *N* random pairs were picked according to the mating probability *p*_*i*_, where selfing was excluded. Second, each parent contributed a single gamete to the offspring wherein crossing over events were introduced according to the specified recombination rate (see above). Third, fitness of the offspring *w*_*i*_ was calculated from the abundance of TE insertions in the resulting genome of the offspring. And fourth, transposition events were introduced according to the transposition rate *v*. Note that the novel TE insertions will only contribute to fitness in the next generation. This could for example be interpreted as TE activity in the germline which will mostly also only effect the next generation (i.e.: the offspring). In all simulations, we performed forward simulations for 10,000 generations. We noted that if a stable equilibrium could be reached (e.g.: no increase in the number of fixed insertions), it took less than 5,000 generations. To match the analysis of natural populations we also sampled 145 haploid genomes after the 10,000 generations and required a minimum count of 3 to identify a TE (see above).

#### Constant population size

In order to estimate the expected variance in TE copy number under an equilibrium model and an constant population size, we performed forward simulations for populations of *N* = 10.000 diploid individuals. We performed 10,427 individual forward simulations with transpositions rates randomly sampled from a uniform distribution between *v* = 0.0–0.003. These simulations required approximately 10,000 CPU hours. Different TE families may have markedly different transposition rates [7] which will result in different equilibrium copy numbers. We therefore identified for every TE family (*j*) the most likely transposition rate *v* that maximizes the probability of observing both the TE copy number of *D. melanogaster* ($c_{j}^{m}$) and of *D. simulans* ($c_{j}^{s}$). To do this, we grouped the simulation results based on the transposition rate *v* into *i* overlapping windows (*W*_*i*_ ∈ *W*) with a window size of 10^−4^ and a step size of 10^−5^ and fitted, for every window, a normal distribution to the data (${𝓝}_{i}(\mu_{i},\sigma_{i}^{2})$ with mean *μ*_*i*_ and standard deviation $\sigma_{i}^{2}$). The probability that a given number of TE insertions (*c*) can be explained by the transposition rate of window *W*_*i*_ is than given by *P*(*c*∣*W*_*i*_) = 1 − *P*(*μ*_*i*_ − ∣*μ*_*i*_ − *c*∣ < *x* < *μ*_*i*_ + ∣*μ*_*i*_ − *c*∣) which can be easily computed from 𝓝_*i*_.

Next we identified for every TE family (*j*) the window ($W_{max}^{j}$) that maximizes the probability of observing $c_{j}^{s}$ and $c_{j}^{m}$ as $W_{max}^{j}={\text{max}}_{W_{i}\in W}[P(c_{j}^{m}|W_{i})P(c_{j}^{s}|W_{i})]$. The corresponding transposition rate of this window will also be the maximum likelihood estimate of *v*. Finally the probability of observing both $c_{j}^{s}$ and $c_{j}^{m}$ with a constant transposition rate as found in window $W_{max}^{j}$ can be computed as $P(c_{j}^{m},c_{j}^{s}|W_{max}^{j})=P(c_{j}^{m}|W_{max}^{j})P(c_{j}^{s}|W_{max}^{j})$. We tested every TE family for significance using Bonferroni correction to account for multiple testings.

#### Varying population size

In order to include demography into our model of TE dynamics we estimated differences in effective population sizes by comparing the level of nucleotide polymorphism in *D. melanogaster* and *D. simulans*. We found that *D. simulans* has a 1.519 higher effective population size than *D. melanogaster*. Accordingly, we performed forward simulations with two different population sizes where the larger population (*N* = 10.000) represents *D. simulans* and the smaller population (*N* = 6,583; ≈ 10000/1.519) represents *D. melanogaster*. Differences in TE insertions between these two species were assessed as described above. The only difference was that, for every window (*i*) we fitted two separate normal distriubtions to the data, one for *D. melanogaster* (${𝓝}_{i}^{m}(\mu_{m,i},\sigma_{m,i}^{2})$) and one for *D. simulans* (${𝓝}_{i}^{s}(\mu_{s,i},\sigma_{s,i}^{2})$). The probability that a given number of TE insertions in *D. melanogaster* (*c* ^*m*^) can be explained by the transposition rate of the given window (*W*_*i*_) can be calculated as $P(c^{m}|W_{i})=1-P((\mu_{m,i}-|\mu_{m,i}-c|<x<\mu_{m,i}+|\mu_{m,i}-c|)|{𝓝}_{i}^{m})$, and accordingly, the probability that the number of TE insertions in *D. simulans* can be explained by the transposition rate in the same window as $P(c^{s}|W_{i})=1-P((\mu_{s,i}-|\mu_{s,i}-c|<x<\mu_{s,i}+|\mu_{s,i}-c|)|{𝓝}_{i}^{s})$. Finally, the maximum likelihood window and the probability of observing both TE counts with the window-specific transposition rate were computed as described above. Again, we used Bonfferoni correction to account for multiple testing.

## Supporting Information

S1 Table A table showing for each TE family the number of mapped reads, the number of paired end reads supporting a TE insertion (one read maps to a TE while the other read maps to a reference chromosomes), and the numbers of identified insertions after various filtering steps. (XLSX)

S2 Table Abundance of all and of fixed TE insertions in *D. melanogaster* and *D. simulans*. Data are shown for the entire genome and the major chromosome arms separately.
(PNG)

S3 Table A table showing for each TE family the average population frequency in both species. (XLSX)

S4 Table Barcodes used in the sequenced Illumina paired-end lanes. (PNG)

S5 Table Mapping statistics for *D. melanogaster*. (PNG)

S6 Table Mapping statistics for *D. simulans*. (PNG)

S7 Table Mapping statistics for *D. simulans* and *D. melanogaster* after subsampling of PE reads to an uniform physical coverage in both species. (PNG)

S1 Fig Relationship between TE expression and population frequency in *D. simulans*. (PDF)

S1 Text Validation of our pipeline for estimating TE abundance. (PDF)

S2 Text Fixed TE insertions are enriched for insertions shared between *D. simulans* and *D. melanogaster*. (PDF)

S3 Text Influence of different simulation parameters on the abundance of TE insertions under drift using an equilbrium model. (PDF)

S4 Text Nucleotide polymorphism in *D. melanogaster* and *D. simulans*. (PDF)
