# Supplementary figures and images for: Tempo and Mode of Transposable Element Activity in Drosophila
Source: PLoS Genet. 2015 Jul 17;11(7):e1005406. doi: 10.1371/journal.pgen.1005406 (PMC4505896; doi:10.1371/journal.pgen.1005406)

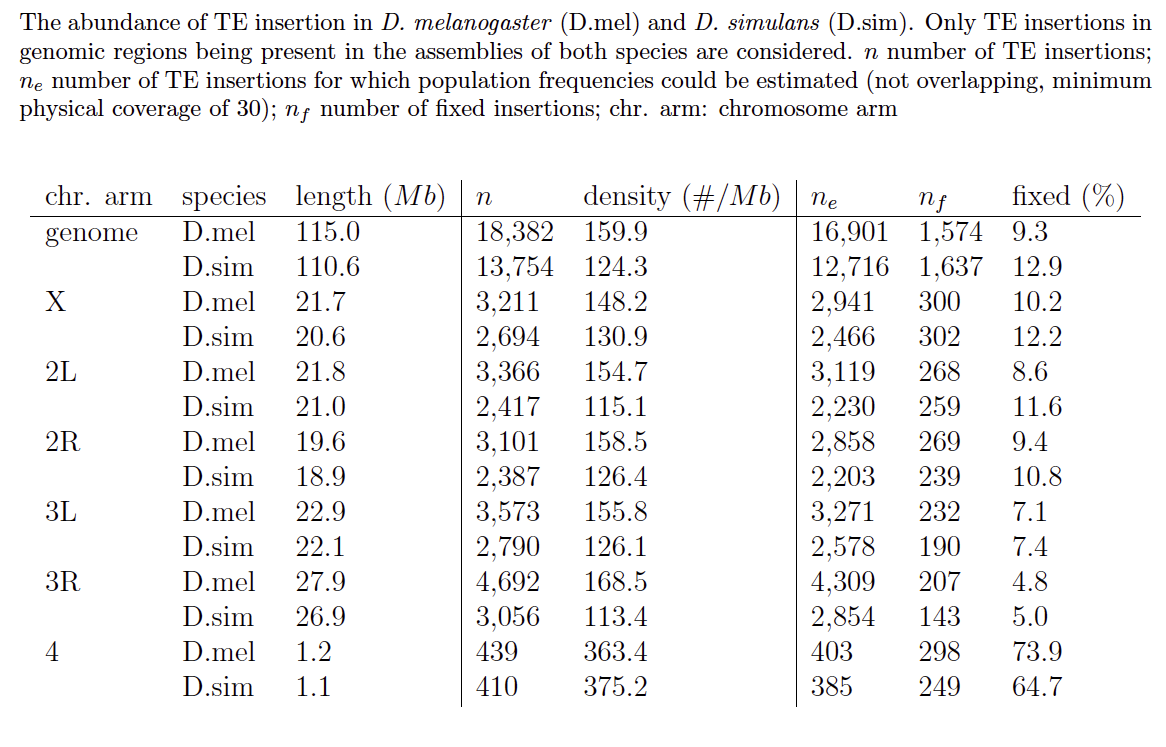

Supplement: S2 Table — Data are shown for the entire genome and the major chromosome arms separately. (PNG) [file pgen.1005406.s002.png]

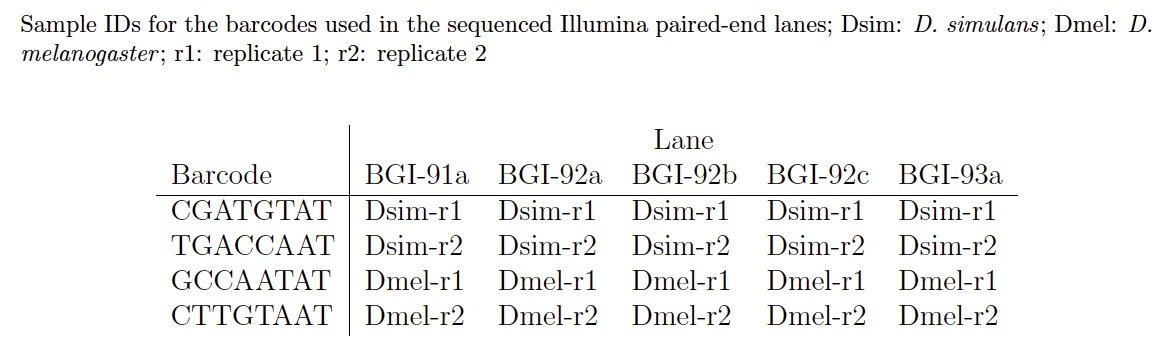

Supplement: S4 Table — (PNG) [file pgen.1005406.s004.png]

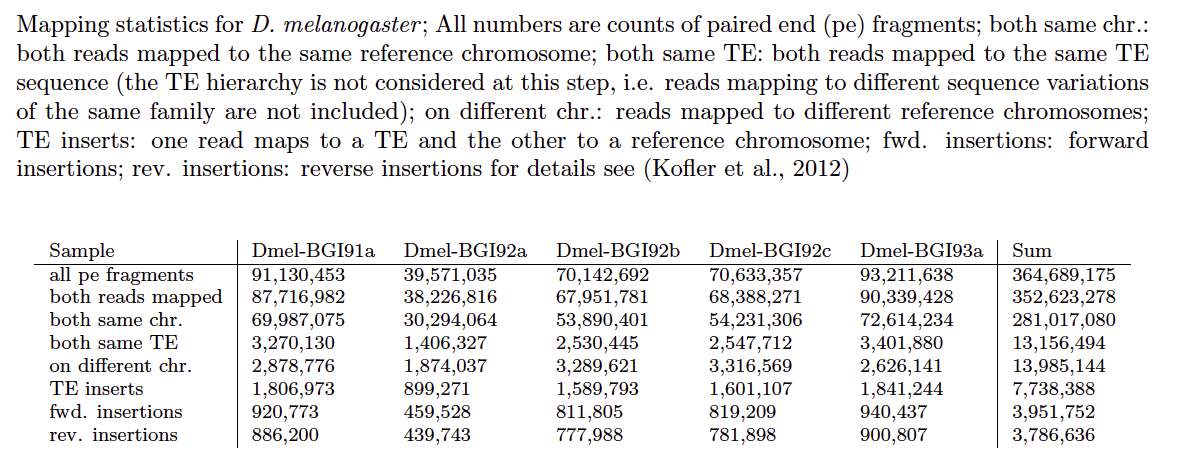

Supplement: S5 Table — (PNG) [file pgen.1005406.s005.png]

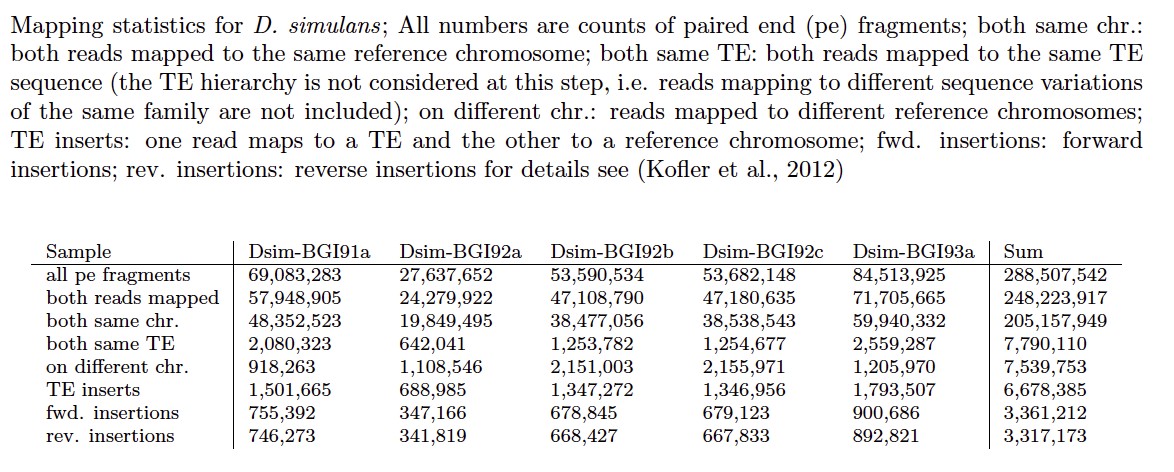

Supplement: S6 Table — (PNG) [file pgen.1005406.s006.png]

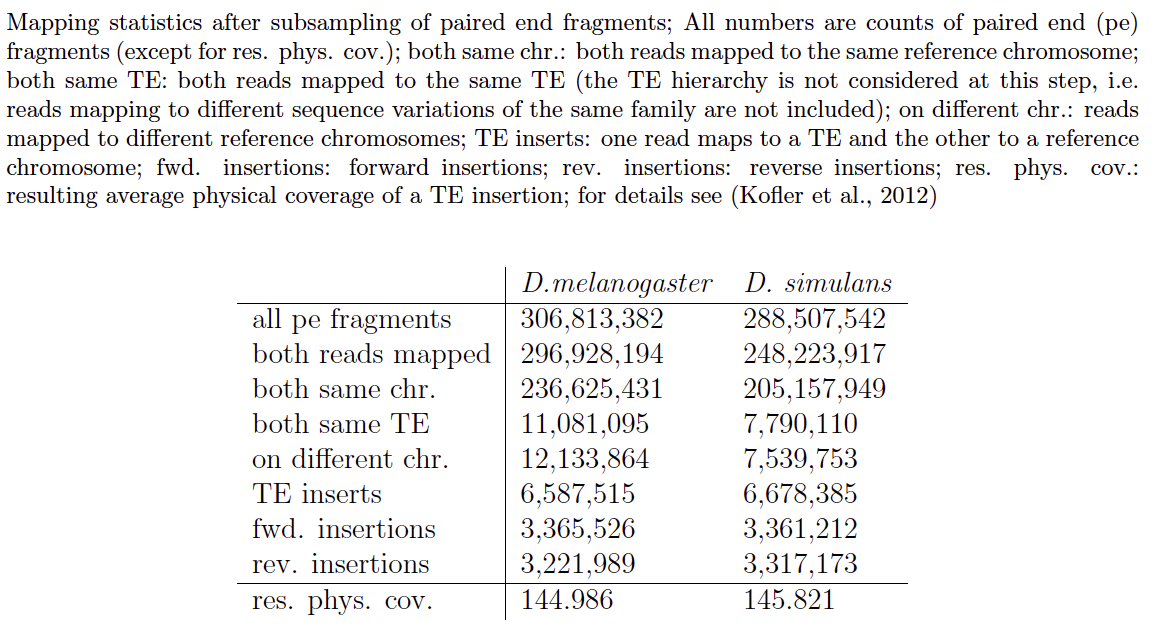

Supplement: S7 Table — (PNG) [file pgen.1005406.s007.png]
