# Supplementary material for: Tempo and Mode of Transposable Element Activity in Drosophila
Source: PLoS Genet. 2015 Jul 17;11(7):e1005406. doi: 10.1371/journal.pgen.1005406 (PMC4505896; doi:10.1371/journal.pgen.1005406)
Supplement: S1 Fig — (PDF) [file pgen.1005406.s008.pdf]

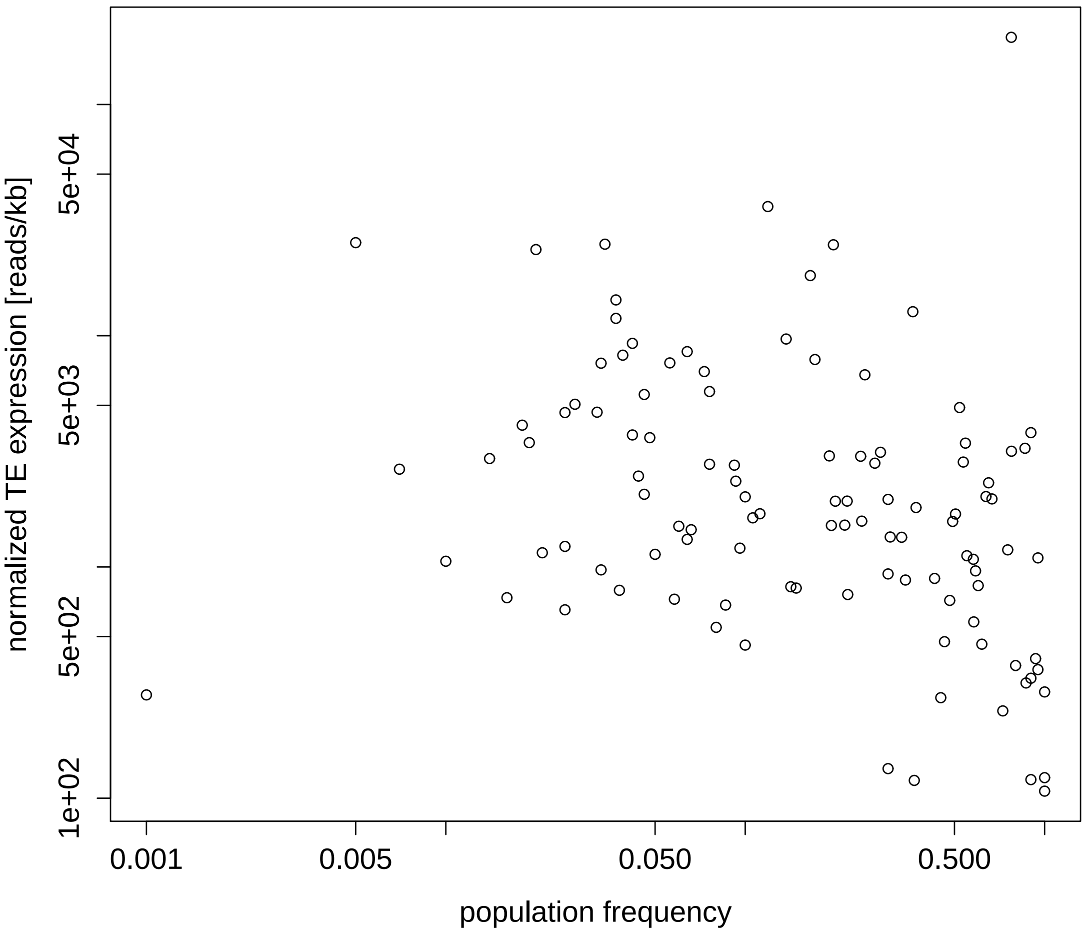

Relationship between normalized TE expression (mapped reads per kb consensus sequence) and population frequency in *D. simulans*. Results are shown for TE families with at least one non-overlapping insertions at a major chromosome arm (111).
