# Supplementary material for: Tempo and Mode of Transposable Element Activity in Drosophila
Source: PLoS Genet. 2015 Jul 17;11(7):e1005406. doi: 10.1371/journal.pgen.1005406 (PMC4505896; doi:10.1371/journal.pgen.1005406)
Supplement: S1 Text — (PDF) [file pgen.1005406.s009.pdf]

Table 1: Performance of the *de novo* annotation pipeline for TE insertions relative to the reference annotation of *D. melanogaster* (*D.mel.* v5.53). The performance was measured at the level of individual insertions using different minimum overlaps (ins.; minimum number of overlapping base pairs is in brackets) and at the nucleotide level (nuc). We estimated the number of true positives (TP) and of false positives (FP). Numbers in brackets are percentages relative to the reference annotation.

	D.mel. v5.53	TP	FP
ins (1 bp).	5,432	4,516 (83.1%)	534 (9.8%)
ins (10 bp).	5,422	4,488 (82.7%)	563 (10.4%)
ins (100 bp).	4,262	4,078 (95.7%)	760 (17.8%)
nuc.	6,556,993	6,266,442 (95.6%)	343,628 (5.2%)

Sensitivity and specificity of the TE annotation pipeline

To test the performance of our pipeline for the *de novo* annotation of TE insertions we used the reference annotation of *D. melanogaster* (FlyBase v5.53) as 'gold standard' and asked whether our pipeline reproduces this reference annotation. We excluded peri-centromeric regions that have, so far, not been annotated for TE insertions (2R:>22,420,241bp, 2L:<387,345bp, 3L:>23,825,333bp; Casey Bergman personal communication). We assessed the performance of our pipeline by estimating the fraction of true positives (TP: reference TE insertions found with our pipeline) and the fraction of false positives (FP: novel TE insertions found with our pipeline, relative to the number of reference insertions). We found that our *de novo* annotation pipeline has a high sensitivity (high fraction of TP) and specificity (low fraction of FP) both at the nucleotide level and the level of individual TE insertions (table 1). In our annotation pipeline we filtered for TE insertions having a minimum length of 100bp (see Material and Methods) and we will thus miss short TE insertions. In agreement with this, the performance of our pipeline increases with an increasing minimum overlap between reference and *de novo* insertions (insertions shorter than the minimum overlap where filtered; table 1)).

Reproducibility of identification of reference insertions

While our *de novo* annotation has a high sensitivity and specificity (section), it is not clear if this will also result in a reliable identification of TE insertions, as our workflow for TE identification (PoPoolation TE; (Kofler et al., 2012)) solely relies on paired end fragments

mapped to TEs and does not necessarily require a TE annotation. A high quality annotation will however serve to improve the performance of our pipeline. To evaluate the impact of our *de novo* annotation on TE identification, we compared the set of reference insertions identified in a *D. melanogaster* population from Portugal (Kofler et al., 2012) with our pipeline using either our *de novo* or the reference annotation [data from Kofler et al. (2012)]. We found that the sets of reference insertions identified with these two approaches are very similar (77%-87%; fig. 1A), especially when short TE insertions (<100bp) are excluded (88%-91%; fig. 1B). Note that it is not expected that all 5,222 TE insertions annotated in the *D. melanogaster* reference genome are also found in the population from Portugal, as most TE insertions segregate at low frequencies (Kofler et al., 2012; Lee and Langley, 2010; Petrov et al., 2011). These results suggests that our *de novo* TE annotation enables a reliable identification of TE insertions, especially for TE insertions longer than 100bp.

Quality control for species pools

We used a very large number of individuals (> 500) from both species to establish isofemale lines and subsequently the pools used for this study. Since *D. melanogaster* and *D. simulans* are phenotypically similar, two different people checked each isofemale line. Since, it is possible that an error occurred in the species identification, we decided to additional check the sequenced pools. We compiled a set of 9,491 SNPs on chromosome 4 that are fixed for different alleles in the two species (R. Tobler, pers. communication). Using these SNPs we found that 0.042% of the base calls in the *D. melanogaster* library are identical to the allele fixed in *D. simulans*, which is close to the fraction of sequencing errors in this library (0.035%). Similarly for the *D. simulans* library we found that 0.014% of the base calls are identical to the allele fixed in *D. melanogaster*, which is again similar to the level of sequencing errors in this library (0.018%). The level of sequencing errors was estimated as the fraction of base calls at these 9,491 SNPs that are neither identical to the allele fixed in *D. melanogaster* nor to the allele fixed in *D. simulans*. We therefore conclude that each of the pools of individuals was derived from a single species only.

Reproducibility of results of Kofler et al. (2012)

We also tested whether estimates of TE abundance generated with our *de novo* TE annotation match previously published results, that were generated with the reference annotation (Kofler et al., 2012). Kofler et al. (2012) estimated the TE abundance in a natural population of *D. melanogaster* from northern Portugal (Povoa de Varzim) using PoPoolation TE and the reference annotation. We compared the estimates of TE abundance obtained in this work with the results of Kofler et al. (2012) and found a good agreement for the population frequency (fig. 2A; Spearman's rank correlation, $r_S = 0.82$, $p < 2.2e - 16$) as well as for the number of insertions (fig. 2B; Spearman's rank correlation, $r_S = 0.81$, $p < 2.2e - 16$). We note that some deviation in the TE abundance between these two samples are expected (Vieira et al., 1999). This good agreement between estimates of TE abundance - despite different annotations, different geographic origins of the populations, different read length (here 100 vs 74), different library preparation methods - suggests that our approach yields highly reliable estimates of TE abundance. In agreement with this, the reliability of PoPoolation TE was recently also confirmed by a simulation study (Zhuang et al., 2014). However, some TE families show marked differences in the numbers of TE insertions between Portugal and South Africa (fig. 2). While the lack of P-element insertions in the population from Portugal can simply be explained by the fact that P-elements were not considered in the study of Kofler et al. (2012), the higher copy numbers of R1A1-elements (South Africa 746; Portugal 11) in South Africa and of gypsy2 (South Africa 14; Portugal 50) elements in Portugal, may be due to different activities of these TE families in the two populations.

Reproducibility of results of Vieira et al. (1999)

Vieira et al. (1999) estimated the average abundance of 36 TE families across multiple populations sampled from a diverse geographic regions (Australia, Europe, Africa, America, Asia) of *D. melanogaster* and *D. simulans* using *in situ* hybridization. In order to enable comparing our data with the results of Vieira et al. (1999), who provided the abundance of TE families as average counts per individual genome, we simply weighted every TE insertion by it's population frequency (population frequency can be interpreted as the probability of observing a given insertion in a random genome). We did not include TE families for which Vieira et al. (1999) and our study, did not find a single insertion (*osvaldo*, *gandalf*,

telemac, *bilbo*), as inclusion of such families may lead to artificially inflated correlations. We also did not include P-element and mariner insertions, as abundance of these two families were not directly estimated by Vieira et al. (1999). Overall we found a striking correlation between TE abundance estimated in this study and the study of Vieira et al. (1999), both for *D. melanogaster* (Spearman's rank correlation; $r_S = 0.85$, $p = 3.6e-9$) and *D. simulans* (Spearman's rank correlation; $r_S = 0.62$, $p = 0.0002$). These correlations are likely conservative estimates as Vieira et al. (1999) sampled populations from many diverse geographic origins while we only analyzed a single population from South Africa. The lower correlation in *D. simulans* may be due to the observed high variability in TE abundance between *D. simulans* populations (Biémont et al., 2003). We note that absolute insertion numbers cannot be compared directly, as Vieira et al. (1999) excluded pericentromeric regions and provided the TE abundance per diploid genome, whereas we only analyzed regions being present in the assemblies of both species and provided the TE abundance per haploid genome.

Table 2: Number of TE insertions per genome for natural populations of *D. melanogaster* (*D.mel.*) and *D. simulans* (*D.sim.*) as estimated by Vieira et al. (1999) and by this study.

	Vieira et al. (1999)		this study	
family	*D.mel.*	*D.sim.*	*D.mel.*	*D.sim.*
1731	1.55	1.00	5.4	5.8
17.6	2.50	0.00	7.3	0.0
297	23.40	1.00	40.5	18.0
412	28.45	13.88	16.0	6.8
BEL/3S18	5.25	0.58	7.7	9.2
blood	17.45	2.5	10.35	4.3
burdock	10.35	5.27	13.0	10.3
copia	24.05	3.88	13.4	0.7
coral/transpac	15.85	1.88	13.8	1.8
flea	16.60	3.42	16.0	9.3
gypsy	1.70	1.54	7.2	1.9
HMS beagle	9.50	2.77	9.3	6.8
idefix	5.70	1.00	10.8	6.3
mdg1	20.75	0.19	16.4	7.3
mdg3	14.10	3.35	7.8	4.4
opus	20.90	4.81	18.1	8.8
prygun/rover	11.35	0.81	4.2	0.9
roo	67.60	38.46	122.6	100.4
springer	2.35	0.00	2.7	1.3
stalker	6.50	0.38	1.7	1.0
tirant	11.45	1.62	7.8	0.9
ZAM	0.35	0.23	1.5	1.7
Doc	26.20	13.81	20.3	7.7
F-element	31.40	1.77	36.3	8.3
helena	0.25	10.23	2.8	12.9
I	25.15	12.58	10.6	33.7
jockey	31.60	3.27	93.7	21.4
bari-1	4.37	4.88	8.2	4.5
hobo	49.90	66.23	29.9	136.4
pogo	13.25	0.00	39.5	0.0

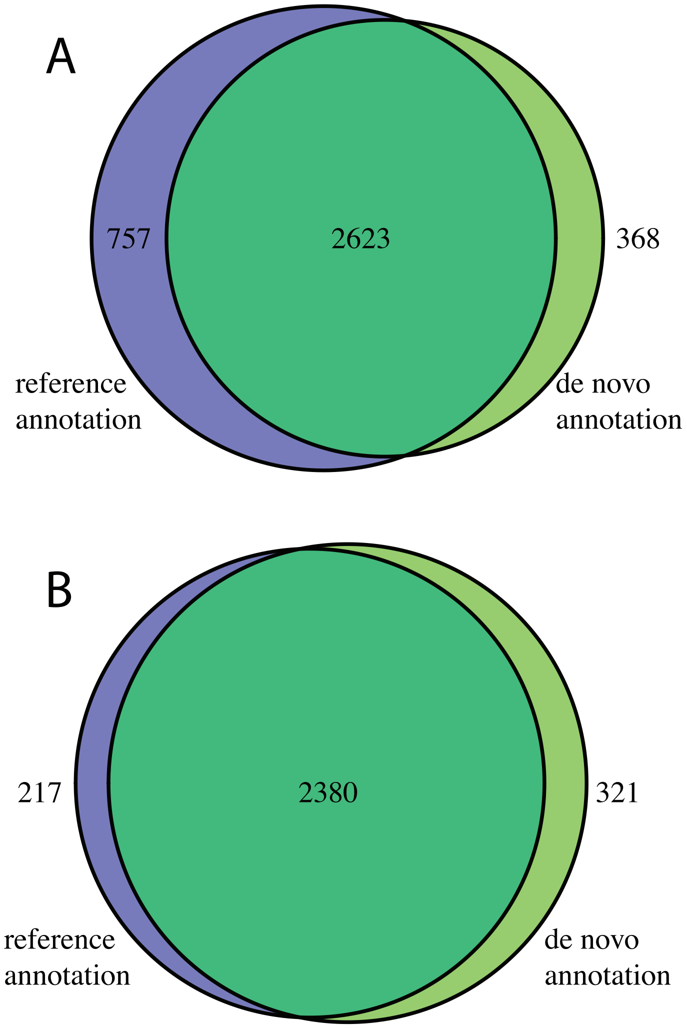

Figure 1: Reference insertions identified in a population from Portugal (Kofler et al., 2012) with PoPoolation TE using either the reference annotation (v5.31) or our *de novo* TE annotation. Results are shown for all reference insertions (A) and reference insertions having a minimum length of 100bp (B).

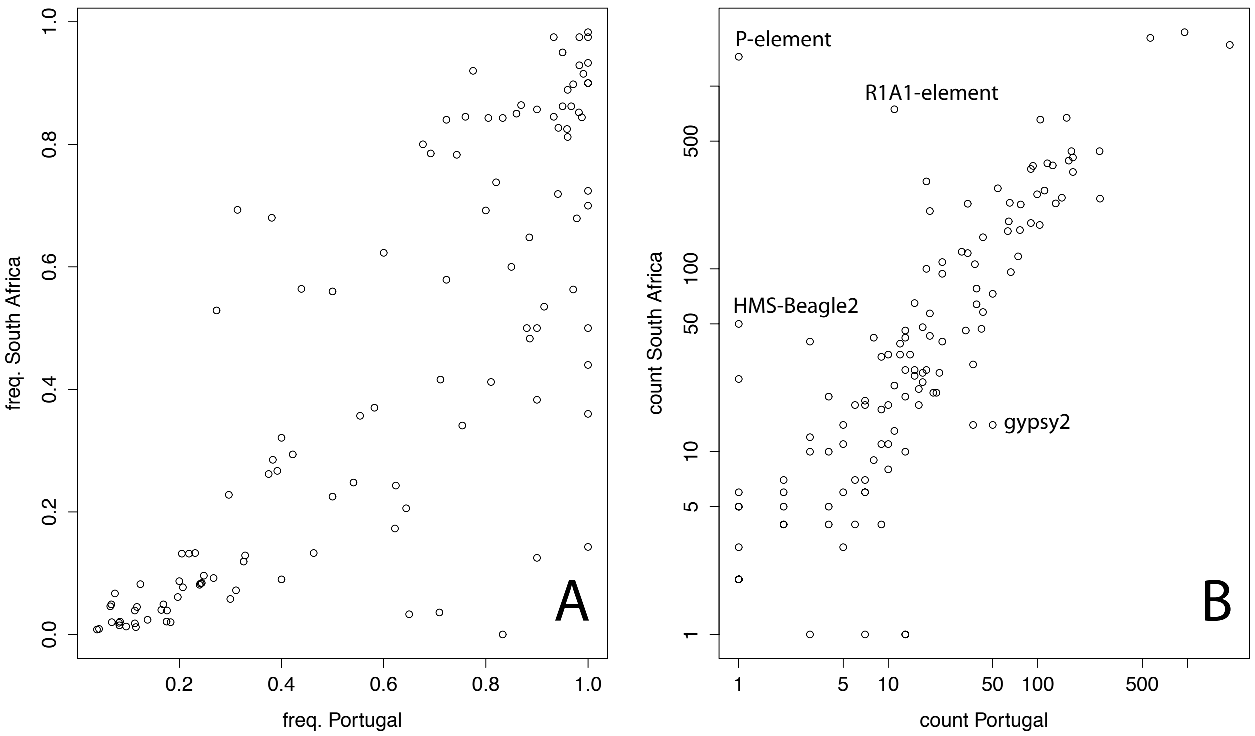

Figure 2: Comparison of the abundance of TE families in two natural populations of *D. melanogaster*: a population from northern Portugal (from Kofler et al. (2012)) and a population from Southern Africa (this work). The average population frequency (A) and the number of insertions (B) are shown. freq.: frequency

References

Biémont, C., Nardon, C., Deceliere, G., and Lepetit, D. (2003). Worldwide distribution of transposable element copy number in natural populations of *Drosophila simulans*. *Evolution*, 57(1):159–167.

Kofler, R., Betancourt, A. J., and Schlötterer, C. (2012). Sequencing of pooled dna samples (pool-seq) uncovers complex dynamics of transposable element insertions in *Drosophila melanogaster*. *PLoS genetics*, 8(1):e1002487.

Lee, Y. C. G. and Langley, C. H. (2010). Transposable elements in natural populations of *Drosophila melanogaster*. *Philosophical transactions of the Royal Society of London. Series B, Biological sciences*, 365(1544):1219–28.

Petrov, D. A., Fiston-Lavier, A.-S., Lipatov, M., Lenkov, K., and González, J. (2011). Population genomics of transposable elements in *Drosophila melanogaster*. *Molecular biology and evolution*, 28(5):1633–44.

Vieira, C., Lepetit, D., Dumont, S., and Biémont, C. (1999). Wake up of transposable elements following *Drosophila simulans* worldwide colonization. *Molecular biology and evolution*, 16(9):1251–5.

Zhuang, J., Wang, J., Theurkauf, W., and Weng, Z. (2014). TEMP: a computational method for analyzing transposable element polymorphism in populations. *Nucleic acids research*.
