# Supplementary material for: Tempo and Mode of Transposable Element Activity in Drosophila
Source: PLoS Genet. 2015 Jul 17;11(7):e1005406. doi: 10.1371/journal.pgen.1005406 (PMC4505896; doi:10.1371/journal.pgen.1005406)
Supplement: S2 Text — (PDF) [file pgen.1005406.s010.pdf]

Age of fixed TE insertions

Reasoning that fixed TE insertions are mostly old, we asked whether fixed insertions are enriched for insertions shared between *D. melanogaster* and *D. simulans*. Shared insertions should mostly predate the split of these two species, which occurred approximately 2-3 million years ago (Hey and Kliman, 1993; Lachaise et al., 1988). To do this, we first generated a set of TE insertions that may potentially be shared between these two species by reciprocally aligning $1000bp$ regions flanking the TE insertion, both at the 5' and the 3' end, to the reference genomes of *D. melanogaster* and *D. simulans* using bwa-sw (v0.7.5a) (Li and Durbin, 2010). We only retained TE insertions (i) where the flanking regions could be unambiguously mapped (mapping quality ≥ 15) and (ii) where the flanking regions could be mapped back to the original positions. This procedure yielded a set of $15,079$ TE insertions that are potentially shared between the two species, i.e.: insertions in non-repetitive regions and insertions in regions that are present in the assemblies of both species (table 1). Actually shared insertions where subsequently identified by scanning for TE insertions of a given family having, in both species, insertion positions within the boundaries of these flanking regions. Note that this procedure allows for some degree of uncertainty in the exact insertion position of the TEs (as for example advisable when using PoPoolation TE). We generated an additional data set excluding the P-element, which has a strong insertion bias (Spradling et al., 2011) that may potentially bias our results. To ensure that any significant enrichment is not solely based on INE-1, a very old TE family that has not been active for >3 million years (Kapitonov and Jurka, 2003; Sackton et al., 2009; Singh and Petrov, 2004), we also generated a data set excluding INE-1 insertions (table 1).

We found that, for all three data sets (table 1), fixed TE insertions are indeed enriched for shared insertions (Fisher's exact test; $p < 2.2e-16$).

Table 1: Numbers of fixed (fix) or segregating (nfix) TE insertions being present either in both species (sh) or just in *D. melanogaster* (nsh). Data are shown for all insertions that could potentially be shared between both species (all), and subsets of these data, excluding either only the P-element (excl. P) or the P-element and INE-1 (excl. P,I).

	all	exlc. P	excl. P,I
fix-sh	577	577	57
fix-nsh	188	188	100
nfix-sh	868	440	315
nfix-nsh	13,446	12,491	12,378
sum	15,079	13,696	12,850

References

Hey, J. and Kliman, R. M. (1993). Population genetics and phylogenetics of DNA sequence variation at multiple loci within the *Drosophila melanogaster* species complex. *Molecular Biology and Evolution*, pages 804–822.

Kapitonov, V. V. and Jurka, J. (2003). Molecular paleontology of transposable elements in the *Drosophila melanogaster* genome. *Proceedings of the National Academy of Sciences of the United States of America*, 100(11):6569–74.

Lachaise, D., Cariou, M. L., David, J. R., and Lemeunier, F. (1988). Historical biogeography of the *Drosophila melanogaster* species subgroup. *Evolutionary Biology*, 22:159–222.

Li, H. and Durbin, R. (2010). Fast and accurate long-read alignment with Burrows-Wheeler transform. *Bioinformatics (Oxford, England)*, 26(5):589–95.

Sackton, T. B., Kulathinal, R. J., Bergman, C. M., Quinlan, A. R., Dopman, E. B., Carneiro, M., Marth, G. T., Hartl, D. L., and Clark, A. G. (2009). Population genomic inferences from sparse high-throughput sequencing of two populations of *Drosophila melanogaster*. *Genome biology and evolution*, 1:449–65.

Singh, N. D. and Petrov, D. A. (2004). Rapid sequence turnover at an intergenic locus in *Drosophila*. *Molecular biology and evolution*, 21(4):670–80.

Spradling, A. C., Bellen, H. J., and Hoskins, R. A. (2011). *Drosophila* P elements preferentially transpose to replication origins. *Proceedings of the National Academy of Sciences of the United States of America*, 108(38):15948–53.
