# Supplementary material for: Tempo and Mode of Transposable Element Activity in Drosophila
Source: PLoS Genet. 2015 Jul 17;11(7):e1005406. doi: 10.1371/journal.pgen.1005406 (PMC4505896; doi:10.1371/journal.pgen.1005406)
Supplement: S3 Text — (PDF) [file pgen.1005406.s011.pdf]

Influence of different simulation parameters to equilibrium copy numbers

The simulations of TE dynamics under an equilibrium model are computationally demanding, where for example a single simulation with a population size $N = 10,000$ for $10,000$ generations requires 1 CPU hour. Thus we aimed to minimize the necessary computation time and performed simulations for 10,000 generations (fig 1A). Neither the mean nor the standard deviation of TE copy numbers at generation 10,000 are significantly different at generation 100,000 ($\bar{x}_{9000<g<1100} = 263.2$, $\bar{x}_{98k<g<100k} = 265.9$, Wilcox ran sum test, $W = 1709.5$, $p = 0.5371$;$s_{9000<g<1100} = 20.13$, $s_{98k<g<100k} = 19.58$,F-test, $F = 1.0569$, $p = 0.83$). The slightly higher standard deviation of TE copy numbers at generation $10,000$ also holds when a larger sample is used ($s_{9000<g<1400} = 19.95$, $s_{95k<g<100k} = 18.23$,F-test, $F = 1.19$, $p = 0.183$) which suggests that performing simulations for 10,000 generations provides a suitable and potentially slightly conservative estimate of TE copy numbers at transposition selection equilibrium. Also for computational reasons we used a population size of $N = 10,000$ in our simulations, rather than estimates of the *D. melanogaster* population size which is assumed to be in the order of several millions (Kreitman, 1983). However, as the standard deviation of equilibrium copy numbers decreases with increasing population size (400 simulations for an average copy number of 600: $s_{N=6583} = 16.54$, $s_{N=10000} = 13.47$, F-test, $F = 0.663$, $p = 4.4e{-}05$; see also supplementary fig 1B) our approach is robust to population sizes larger than 10,000. *D. simulans* has a about 1.3 times longer genetic map than *D. melanogaster* (True et al., 1996) and consequently also a higher recombination rate. Such differences in recombination rates may influence equilibrium copy numbers of TE insertions (Dolgin and Charlesworth, 2008). As estimates of the recombination rates for genomic loci on all major chromosome arms are only available for *D. melanogaster* we investigated the influence of the recombination rate by simply scaling the recombination map of *D. melanogaster* by the factor 1.3 using the Kosambi function. As the standard deviation of equilibrium copy numbers decreases with recombination rate (400 simulations with $N = 6583$ and an average copy number of 292; rr=recombination rate; $s_{rr=Dmel} = 28.35$, $s_{rr=1.3*Dmel} = 23.68$, F-test $F = 0.697$, $p = 0.00033$) our simulation results are robust for recombination rates higher than in *D. melanogaster*. Furthermore, we found that increasing the recombination rate 1.3 fold (as in *D. simulans*) has a much small effect to equilibrium copy numbers

than increasing the population size 1.519 fold (as in *D. simulans*; $\bar{x}_{N=6583,rr=Dmel} = 292.66$, $\bar{x}_{N=10000,rr=Dmel} = 263.57$, $\bar{x}_{N=6583,rr=1.3*Dmel} = 291.73$, Wilcox rank sum test, $W = 32916$, $p < 2.2e - 16$), therefore our simulations with two different population sizes also implicitly cover the case of two different recombination rates, albeit not both (different population sizes and recombination rates) at the same time. Increasing the degree of synergistic effect of TE insertions (400 simulations for $t = 1.3$ and 106 for $t = 1.75$ for equilibrium copy number 20; $s_{t=1.3} = 5.52$, $s_{t=1.75} = 4.43$, F-test, $F = 1.55$, $p = 0.0072$) and the selective impact of TE insertions (400 simulations for $x = 0.0004$ and 112 for $x = 0.0010$ for equilibrium copy number 25.95; $s_{x=0.0004} = 6.52$, $s_{x=0.0010} = 5.21$, F-test, $F = 0.641$, $p = 0.00539$) both result in a lower variance of equilibrium copy numbers, our results are therefore robust for $t \geq 1.3$ and $x \geq 0.0004$.

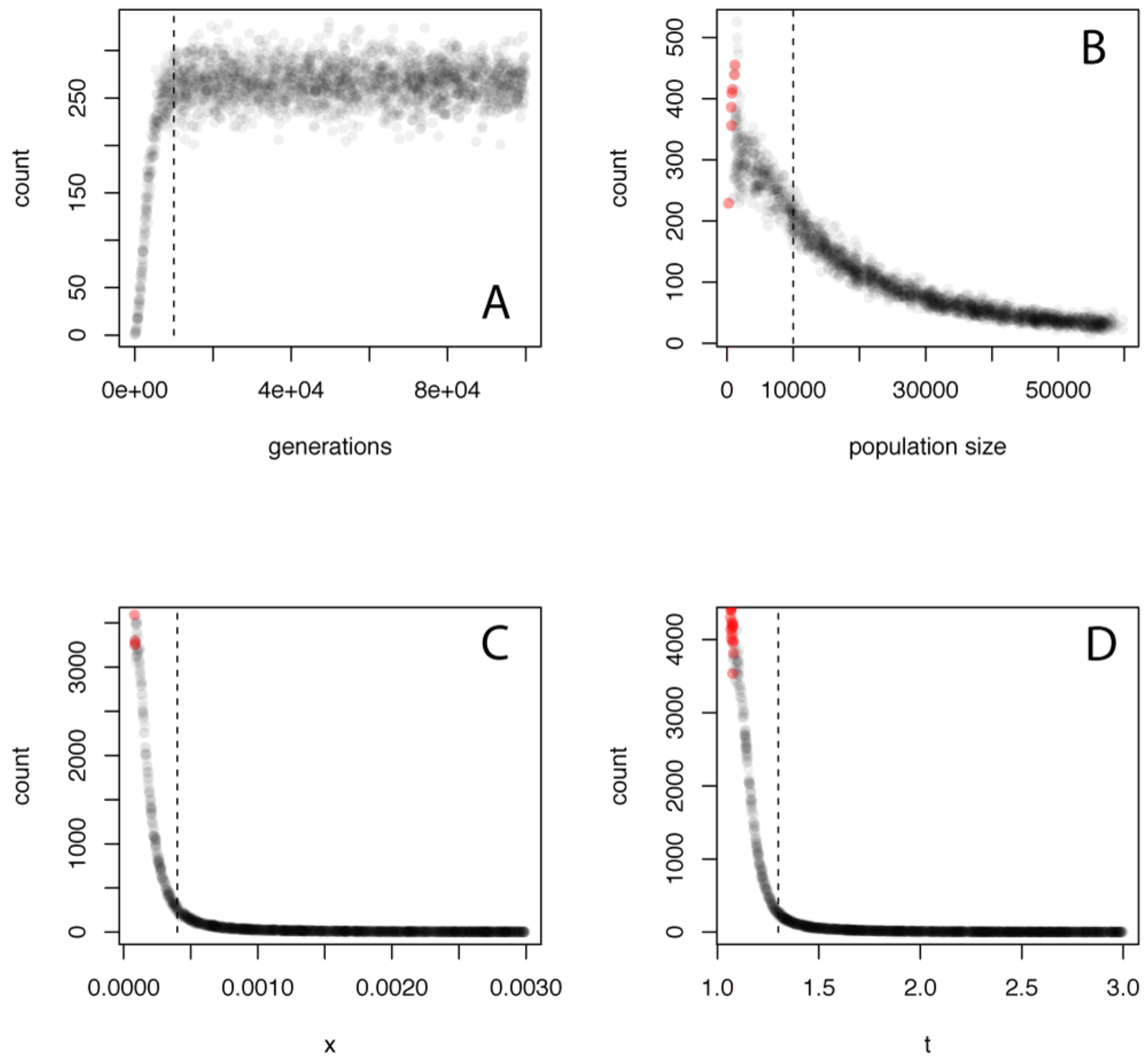

Figure 1: Influence of main parameters on simulation results. A) generations B) population size (N) C) selective impact of a TE insertion (x) D) degree of synergistic effect between insertions (t). Dashed lines represent default parameters of the simulations. Populations for which the average fitness fell below 0.25 were assumed extinct and marked (red dots). Insertion numbers are shown one generation before extinction. We performed 2000 independent simulations for each graph, used consistent default parameters and always only varied the parameter of interest. The following default parameters were used: $v = 0.0015$, $N = 10000$, $x = 0.0004$, $t = 1.3$, $generations = 10,000$.

References

Dolgin, E. S. and Charlesworth, B. (2008). The effects of recombination rate on the distribution and abundance of transposable elements. *Genetics*, 178(4):2169–2177.

Kreitman, M. (1983). Nucleotide polymorphism at the alcohol dehydrogenase locus of *Drosophila melanogaster*. *Nature*, 304(5925):412–417.

True, J. R., Mercer, J. M., and Laurie, C. C. (1996). Differences in crossover frequency and distribution among three sibling species of drosophila. *Genetics*, 142(2):507–523.
