# Supplementary material for: Tempo and Mode of Transposable Element Activity in Drosophila
Source: PLoS Genet. 2015 Jul 17;11(7):e1005406. doi: 10.1371/journal.pgen.1005406 (PMC4505896; doi:10.1371/journal.pgen.1005406)
Supplement: S4 Text — (PDF) [file pgen.1005406.s012.pdf]

Table 1: Nucleotide polymorphism in the natural *D. melanogaster* (*D.mel.*) and *D. simulans* (*D.sim.*) populations. From genomic regions being present in both assemblies (both assemblies) we successively removed low recombining regions (<1 cM/Mb; excl. low. rec) and regions overlapping with exons (excl. coding).

	both assembl.		excl. low. rec.		excl. coding	
	D.mel.	*D.sim.*	*D.mel.*	*D.sim.*	*D.mel.*	*D.sim.*
whole genome	0.0061	0.0104	0.0068	0.0108	0.0070	0.0107
X	0.0052	0.0077	0.0053	0.0082	0.0056	0.0086
2L	0.0072	0.0115	0.0080	0.0121	0.0081	0.0119
2R	0.0063	0.0108	0.0070	0.0109	0.0070	0.0107
3L	0.0067	0.0116	0.0078	0.0118	0.0080	0.0117
3R	0.0055	0.0108	0.0064	0.0112	0.0067	0.0108
4	0.0012	0.0014	-	-	-	-
autosomes	0.0063	0.0110	0.0072	0.0115	0.0074	0.0113

Nucleotide polymorphism

We estimated the nucleotide polymorphism in the natural *D. melanogaster* and *D. simulans* populations with a sliding window approach, using non-overlapping windows of 1 kb (see material and methods in main manuscript). Subsequently we (i) filtered for regions being present in both assemblies, (ii) excluded regions with low recombination rates ($< 1\ cM/Mb$) in *D. melanogaster* and (iii) excluded windows overlapping with an exon (Table 1). Based on the level of polymorphism in the autosomes we estimate that the N_e of *D. simulans* is approximately 1.519 (= 0.011285/0.007429) times higher than the N_e of *D. melanogaster*.
